# Identification of magnetic interactions and high-field quantum spin liquid in *α*-RuCl_3_

**DOI:** 10.1038/s41467-021-24257-8

**Published:** 2021-06-29

**Authors:** Han Li, Hao-Kai Zhang, Jiucai Wang, Han-Qing Wu, Yuan Gao, Dai-Wei Qu, Zheng-Xin Liu, Shou-Shu Gong, Wei Li

**Affiliations:** 1 School of Physics, Beihang University, Beijing, China; 2 Institute for Advanced Study, Tsinghua University, Beijing, China; 3 Department of Physics, Renmin University of China, Beijing, China; 4 Center for Neutron Science and Technology, School of Physics, Sun Yat-sen University, Guangzhou, China; 5 International Research Institute of Multidisciplinary Science, Beihang University, Beijing, China; 6 Institute of Theoretical Physics, Chinese Academy of Sciences, Beijing, China

**Keywords:** Magnetic properties and materials, Phase transitions and critical phenomena, Quantum fluids and solids

## Abstract

The frustrated magnet *α*-RuCl_3_ constitutes a fascinating quantum material platform that harbors the intriguing Kitaev physics. However, a consensus on its intricate spin interactions and field-induced quantum phases has not been reached yet. Here we exploit multiple state-of-the-art many-body methods and determine the microscopic spin model that quantitatively explains major observations in *α*-RuCl_3_, including the zigzag order, double-peak specific heat, magnetic anisotropy, and the characteristic M-star dynamical spin structure, etc. According to our model simulations, the in-plane field drives the system into the polarized phase at about 7 T and a thermal fractionalization occurs at finite temperature, reconciling observations in different experiments. Under out-of-plane fields, the zigzag order is suppressed at 35 T, above which, and below a polarization field of 100 T level, there emerges a field-induced quantum spin liquid. The fractional entropy and algebraic low-temperature specific heat unveil the nature of a gapless spin liquid, which can be explored in high-field measurements on *α*-RuCl_3_.

## Abstract (web summary)

The nature of spin interactions and the field-induced quantum spin liquid phase in the Kitaev material *α*-RuCl_3_ have been debated. Here, using a combination of many-body techniques, the authors derive an effective spin model that explains the majority of experimental findings and predicts a new quantum spin liquid phase in strong out-of-plane magnetic field.

## Introduction

The spin-orbit magnet *α*-RuCl_3_, with edge-sharing RuCl_6_ octahedra and a nearly perfect honeycomb plane, has been widely believed to be a correlated insulator with the Kitaev interaction^1–6^. The compound *α*-RuCl_3_, and the Kitaev materials in general, have recently raised great research interest in exploring the inherent Kitaev physics^7–11^, which can realize non-Abelian anyon with potential applications in topological quantum computations^12,13^. Due to additional non-Kitaev interactions in the material, *α*-RuCl_3_ exhibits a zigzag antiferromagnetic (AF) order at sufficiently low temperature (*T*_c_ ≃ 7 K)^2,14,15^, which can be suppressed by an external in-plane field of 7-8 T^16–18^. Surprisingly, the thermodynamics and the unusual excitation continuum observed in the inelastic neutron scattering (INS) measurements suggest the presence of fractional excitations and the proximity of *α*-RuCl_3_ to a quantum spin liquid (QSL) phase^14,15,19^. Furthermore, experimental probes including the nuclear magnetic resonance (NMR)^17,20,21^, Raman scattering^22^, electron spin resonance (ESR)^23^, THz spectroscopy^24,25^, and magnetic torque^26,27^, etc, have been employed to address the possible Kitaev physics in *α*-RuCl_3_ from all conceivable angles. In particular, the unusual (even half-integer quantized) thermal Hall signal was observed in a certain temperature and field window^28–31^, suggesting the emergent Majorana fractional excitations. However, significant open questions remain to be addressed: whether the in-plane field in *α*-RuCl_3_ induces a QSL ground state that supports the spin-liquid signals in experiment, and furthermore, is there a QSL phase induced by fields along other direction?

To accommodate the QSL states in quantum materials like *α*-RuCl_3_, realization of magnetic interactions of Kitaev type plays a central role. Therefore, the very first step toward the precise answer to above questions is to pin down an effective low-energy spin model of *α*-RuCl_3_. As a matter of fact, people have proposed a number of spin models with various couplings^19,32–42^, yet even the signs of the couplings are not easy to determine and currently no single model can simultaneously cover the major experimental observations^43^, leaving a gap between theoretical understanding and experimental observations. In this work, we exploit multiple accurate many-body approaches to tackle this problem, including the exponential tensor renormalization group (XTRG)^44,45^ for thermal states, the density matrix renormalization group (DMRG) and variational Monte Carlo (VMC) for the ground state, and the exact diagonalization (ED) for the spectral properties. Through large-scale calculations, we determine an effective Kitaev-Heisenberg-Gamma-Gamma$^{\prime}$ (*K*-*J*-Γ-${{\Gamma }}^{\prime}$) model [cf. Eq. (1) below] that can perfectly reproduce the major experimental features in the equilibrium and dynamic measurements.

Specifically, in our *K*-*J*-Γ-${{\Gamma }}^{\prime}$ model the Kitaev interaction *K* is much greater than other non-Kitaev terms and found to play the predominant role in the intermediate temperature regime, showing that *α*-RuCl_3_ is indeed in close proximity to a QSL. As the compound, our model also possesses a low-*T* zigzag order, which is melted at about 7 K. At intermediate energy scale, a characteristic M star in the dynamical spin structure is unambiguously reproduced. Moreover, we find that in-plane magnetic field suppresses the zigzag order at around 7 T, and drives the system into a trivial polarized phase. Nevertheless, even above the partially polarized states, our finite-temperature calculations suggest that *α*-RuCl_3_ could have a fractional liquid regime with exotic Kitaev paramagnetism, reconciling previous experimental debates. We put forward proposals to explore the fractional liquid in *α*-RuCl_3_ via thermodynamic and spin-polarized INS measurements. Remarkably, when the magnetic field is applied perpendicular to the honeycomb plane, we disclose a QSL phase driven by high fields, which sheds new light on the search of QSL in Kitaev materials. Furthermore, we propose experimental probes through magnetization and calorimetry^46^ measurements under 100-T class pulsed magnetic fields^47,48^.

## Results

### Effective spin model and quantum many-body methods

We study the *K*-*J*-Γ-${{\Gamma }}^{\prime}$ honeycomb model with the interactions constrained within the nearest-neighbor sites, i.e.,

1 $$\begin{array}{ll}H=&\mathop{\sum}\limits_{{\langle i,j\rangle }_{\gamma }}\left[K{S}_{i}^{\gamma }{S}_{j}^{\gamma }+J\ {{\bf{S}}}_{i}\cdot {{\bf{S}}}_{j}+{{\Gamma }}\big({S}_{i}^{\alpha }{S}_{j}^{\beta }+{S}_{i}^{\beta }{S}_{j}^{\alpha }\big)\right.\\ &\kern-1.5pc\left.+\,{{\Gamma }}^{\prime} \big({S}_{i}^{\gamma }{S}_{j}^{\alpha }+{S}_{i}^{\gamma }{S}_{j}^{\beta }+{S}_{i}^{\alpha }{S}_{j}^{\gamma }+{S}_{i}^{\beta }{S}_{j}^{\gamma }\big)\right],\end{array}$$

where ${{\bf{S}}}_{i}=\{{S}_{i}^{x},{S}_{i}^{y},{S}_{i}^{z}\}$ are the pseudo spin-1/2 operators at site *i*, and 〈*i*, *j*〉_*γ*_ denotes the nearest-neighbor pair on the *γ* bond, with {*α*, *β*, *γ*} being {*x*, *y*, *z*} under a cyclic permutation. *K* is the Kitaev coupling, Γ and ${{\Gamma }}^{\prime}$ the off-diagonal couplings, and *J* is the Heisenberg term. The symmetry of the model, besides the lattice translation symmetries, is described by the finite magnetic point group ${D}_{{\rm{3d}}}\times {Z}_{2}^{T}$, where ${Z}_{2}^{T}=\{E,T\}$ is the time-reversal symmetry group and each element in *D*_3d_ stands for a combination of lattice rotation and spin rotation due to the spin-orbit coupling. The symmetry group restricts the physical properties of the system. For instance, the Landé *g* tensor and the magnetic susceptibility tensor, should be uni-axial.

**Fig. 1:**
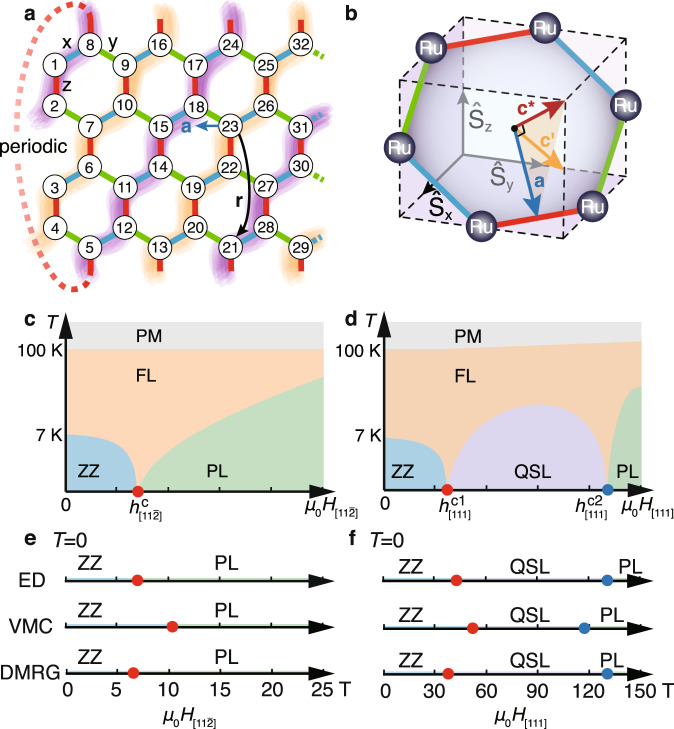
Honeycomb lattice, crystalline directions, and phase diagrams. **a** Shows the YC *W* × *L* × 2 honeycomb lattice of width *W* = 4 and length *L*, with two sites per unit cell. The quasi-1D mapping path and site ordering is shown by the numbers, with the edge of each small hexagon set as length unit. In the zigzag magnetic order, spins align parallel along the highlighted lines, while anti-parallel between the yellow and purple lines. **b** shows an unit cell in *α*-RuCl_3_, with the **a**, c^*^, and ${\bf{c}}^{\prime}$ axes indicated. Finite-temperature phase diagrams under in-plane (${H}_{[11\bar{2}]}\parallel {\bf{a}}$) and out-of-plane (*H*_[111]_∥c^*^) fields are presented in (**c**) and (**d**), respectively, with in-plane critical field ${h}_{[11\bar{2}]}^{c}\simeq 7$ T (computed at 1.9 K), and two out-of-plane critical fields ${h}_{[111]}^{c1}\approx 35$ T (lower) and ${h}_{[111]}^{c2}\approx 120$-130 T (upper), estimated at 0 K. In the phase diagrams, ZZ stands for the zigzag phase, PM for paramagnetic, FL for fractional liquid, and PL for the polarized phase, with the QPTs (red and blue points) indicated. **e**, **f** show the *T* = 0 phase diagrams obtained by ED, VMC and DMRG, where the in-plane critical field is pinpointed at between 6.5 and 10 T, while two QPTs and a QSL phase are uncovered under out-of-plane fields.

We recall that the ${{\Gamma }}^{\prime}$ term is important for stabilizing the zigzag magnetic order at low temperature in the extended ferromagnetic (FM) Kitaev model with *K* < 0^36,49,50^. While the zigzag order can also be induced by the third-neighbor Heisenberg coupling *J*_3_^32,33,51^, we constrain ourselves within a minimal *K*-*J*-Γ-${{\Gamma }}^{\prime}$ model in the present study and leave the discussion on the *J*_3_ coupling in the Supplementary Note 1. In the simulations of *α*-RuCl_3_ under magnetic fields, we mainly consider the in-plane field along the $[11\bar{2}]$ direction, ${H}_{[11\bar{2}]}\parallel {\bf{a}}$, and the out-of-plane field along the [111] direction, *H*_[111]_∥c^*^, with the corresponding Landé factors ${g}_{{\rm{ab}}}(={g}_{[11\bar{2}]})$ and ${g}_{{\rm{{c}}^{* }}}(={g}_{[111]})$, respectively. The index [*l*, *m*, *n*] represents the field direction in the spin space depicted in Fig. 1b. Therefore, the Zeeman coupling between field *H*_[*l*, *m*, *n*]_ to local moments can be written as ${H}_{{\rm{Zeeman}}}={g}_{[l,m,n]}{\mu }_{{\rm{B}}}{\mu }_{0}{H}_{[l,m,n]}{S}_{i}^{[l,m,n]}$, where *S*^[*l*, *m*, *n*]^ ≡ **S** ⋅ **d**_*l*,*m*,*n*_ with **S** = (*S*_*x*_, *S*_*y*_, *S*_*z*_) and ${{\bf{d}}}_{l,m,n}={(l,m,n)}^{T}/\sqrt{{l}^{2}+{m}^{2}+{n}^{2}}$. The site index *i* = 1, ⋯ , *N*, with *N* ≡ *W* × *L* × 2 the total site number.

In the simulations, various quantum many-body calculation methods have been employed (see Methods). The thermodynamic properties under zero and finite magnetic fields are computed by XTRG on finite-size systems (see, e.g, YC4 systems shown in Fig. 1a). The model parameters are pinpointed by fitting the XTRG results to the thermodynamic measurements, and then confirmed by the ground-state magnetization calculations by DMRG with the same geometry and VMC on an 8 × 8 × 2 torus. Moreover, the ED calculations of the dynamical properties are performed on a 24-site torus, which are in remarkable agreement to experiments and further strengthen the validity and accuracy of our spin model. Therefore, by combining these cutting-edge many-body approaches, we explain the experimental observations from the determined effective spin Hamiltonian, and explore the field-induced QSL in *α*-RuCl_3_ under magnetic fields.

### Model parameters

**Fig. 2:**
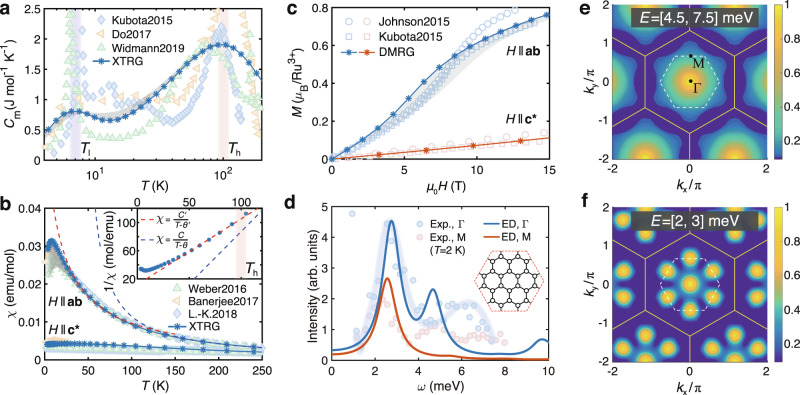
Model simulations of thermodynamic and dynamic properties. With the *K*-*J*-Γ-${{\Gamma }}^{\prime}$ model, we perform many-body simulations and compare the results to experiments, including (**a**) the magnetic specific heat *C*_m_^3,15,52^, (**b**) in-plane (*χ*_ab_) and out-of-plane (${\chi }_{{\rm{{c}}^{* }}}$) susceptibilities (measured at the field of 1 T)^14,53,54^, and (**c**) magnetization curves *M*(*H*)^3,55^ on the YC4 × *L* × 2 lattice, with the gray shaded region indicating the influences of different system lengths *L* = 4, 6. The Curie-Weiss fittings of the high- and intermediate-*T* susceptibility *χ*_ab_ in (**b**) lead to *C* ≃ 0.67 cm^3^K/mol, *θ* ≃ 41.4 K, and $C^{\prime} \simeq 1.07$ cm^3^ K/mol, $\theta ^{\prime} \simeq -12.7$ K, respectively. We also compute the dynamical spin structure by ED and compare the results with neutron scattering intensity ${\mathcal{I}}({\bf{k}},\omega )$ at **k** = Γ and M in (**d**), on a *C*_3_ symmetric 24-site cluster shown in the inset (see more information and its comparison to another 24-site cluster with lower symmetry in the Supplementary Fig. 4). The light blue bold line is a guide to the eye for INS data at Γ point^18^. The calculated intensity peak positions *ω*_Γ_ and *ω*_M_ are in very good agreement with experiments. We further plot the integrated intensities within the energy interval (**e**) [4.5, 7.5] meV and (**f**) [2, 3] meV, where a clear M-star shape is reproduced in (**e**) at intermediate energy and the bright M points are evident in (**f**) at low energy, both in excellent agreement with the INS experiments^14^. The dashed white hexagon marks the first BZ, and the outer yellow hexagon is the extended BZ.

As shown in Fig. 2a-b, through simulating the experimental measurements, including the magnetic specific heat and both in- and out-of-plane susceptibility data^3,14,15,52–55^, we accurately determine the parameters in the Hamiltonian Eq. (1), which read *K* = − 25 meV, Γ = 0.3∣*K*∣, ${{\Gamma }}^{\prime} =-0.02\,| K|$, and *J* = − 0.1∣*K*∣. The in- and out-of-plane Landé factors are found to be *g*_ab_ = 2.5 and ${g}_{{\rm{{c}}^{* }}}=2.3$, respectively. We find that both the magnetic specific heat *C*_m_ and the two susceptibilities (in-plane *χ*_ab_ and out-of-plane ${\chi }_{{\rm{{c}}^{* }}}$) are quite sensitive to the Γ term, and the inclusion of ${{\Gamma }}^{\prime}$(*J*) term can significantly change the low-*T C*_m_(*χ*_ab_) data. Based on these observations, we accurately pinpoint the various couplings. The details of parameter determination, with comparisons to the previously proposed candidate models can be found in Supplementary Notes 1, 2. To check the robustness and uniqueness of the parameter fittings, we have also performed an automatic Hamiltonian searching^56^ with the Bayesian optimization combined large-scale thermodynamics solver XTRG, and find that the above effective parameter set indeed locates within the optimal regime of the optimization (Supplementary Note 1). In addition, the validity of our *α*-RuCl_3_ model is firmly supported by directly comparing the model calculations to the measured magnetization curves in Fig. 2c and INS measurements in Fig. 2d–f.

In our *K*-*J*-Γ-${{\Gamma }}^{\prime}$ model of *α*-RuCl_3_, we see a dominating FM Kitaev interaction and a sub-leading positive Γ term (Γ > 0), which fulfill the interaction signs proposed from recent experiments^6,34,39^ and agree with some ab initio studies^9,33,36,37,43,57^. The strong Kitaev interaction seems to play a predominant role at intermediate temperature, which leads to the fractional liquid regime and therefore naturally explains the observed proximate spin liquid behaviors^14,15,19^.

### Magnetic specific heat and two-temperature scales

We now show our simulations of the *K*-*J*-Γ-${{\Gamma }}^{\prime}$ model and compare the results to the thermodynamic measurements. In Fig. 2a, the XTRG results accurately capture the prominent double-peak feature of the magnetic specific heat *C*_m_, i.e., a round high-*T* peak at *T*_h_ ≃ 100 K and a low-*T* one at *T*_l_ ≃ 7 K. As *T*_h_ ≃ 100 K is a relatively high-temperature scale where the phonon background needs to be carefully deal with^52^, and there exists quantitative difference among the various *C*_m_ measurements in the high-*T* regime^3,15,52^. Nevertheless, the high-*T* scale *T*_h_ itself is relatively stable, and in Fig. 2a our XTRG result indeed exhibits a high-*T* peak centered at around 100 K, in good agreement with various experiments. Note that the high-temperature crossover at *T*_h_ corresponds to the establishment of short-range spin correlations, which can be ascribed to the emergence of itinerant Majorana fermions^15,58^ in the fractional liquid picture that we will discuss. Such a crossover can also be observed in the susceptibilities, which deviate the high-*T* Curie-Weiss law and exhibit an intermediate-*T* Curie-Weiss scaling below *T*_h_^59^, as shown in Fig. 2b for *χ*_ab_ (the same for ${\chi }_{{\rm{{c}}^{* }}}$).

**Fig. 3:**
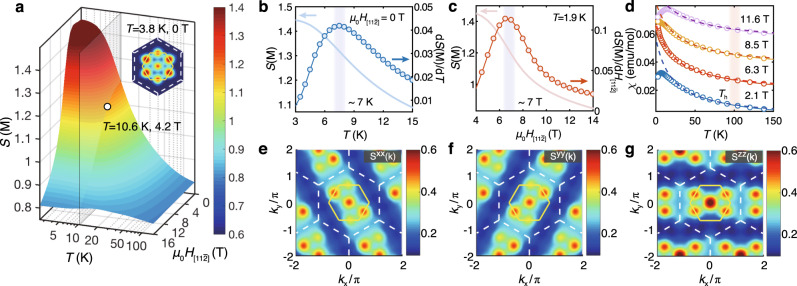
Low-temperature zigzag order and intermediate-*T* fractional liquid regime under in-plane fields ${H}_{[11\bar{2}]}\parallel {\bf{a}}$. **a** Surface plot of the M-point spin structure factor *S*(M) (average over six M points in the BZ) vs. temperature *T* and field ${H}_{[11\bar{2}]}$. The inset is a low-*T* structure factor with six bright M points in the BZ, representing the zigzag order. The *S*(M) curve and its derivative over *T* and ${H}_{[11\bar{2}]}$ are shown in (**b**) and (**c**), respectively, which indicate a suppression of zigzag order at around the temperature *T*_c_ ≃ 7 K and field ${h}_{[11\bar{2}]}^{c}\simeq 7$ T. **d** Shows the emergent Curie-Weiss behavior in the magnetic susceptibility at intermediate *T*, with the fitted $C^{\prime} \simeq$ 1.17, 1.20, 1.21, and 1.27 cm^3^ K/mol and $\theta ^{\prime} \simeq$ −20.7, −24.2, −26, and −34.4 K for fields ${\mu }_{0}{H}_{[11\bar{2}]}=$ 2.1, 6.3, 8.5 and 11.6 T, respectively. The different *χ* curves under various fields are shifted vertically by 0.018 emu/mol for clarify. The bright stripes of spin-resolved magnetic structure factors, i.e., *S*^*xx*^(**k**), *S*^*yy*^(**k**), and *S*^*zz*^(**k**) as shown in (**e**), (**f**), (**g**), respectively, calculated at ${\mu }_{0}{H}_{[11\bar{2}]}=4.2$ T and *T* = 10.6 K, rotate counterclockwise (by 120^∘^) as the spin component switches, reflecting the peculiar bond-oriented spin correlations in the intermediate fractional liquid regime.

At the temperature *T*_l_ ≃ 7 K, the experimental *C*_m_ curves of *α*-RuCl_3_ exhibit a very sharp peak, corresponding to the establishment of a zigzag magnetic order^2,3,14,15,52^. Such a low-*T* scale can be accurately reproduced by our model calculations, as shown in Fig. 2a. As our calculations are performed on the cylinders of a finite width, the height of the *T*_l_ peak is less prominent than experiments, as the transition in the compound *α*-RuCl_3_ may be enhanced by the inter-layer couplings. Importantly, the location of *T*_l_ fits excellently to the experimental results. Below *T*_l_ our model indeed shows significantly enhanced zigzag spin correlation, which is evidenced by the low-energy dynamical spin structure in Fig. 2f and the low-*T* static structure in the inset of Fig. 3a.

### Anisotropic susceptibility and magnetization curves

It has been noticed from early experimental studies of *α*-RuCl_3_ that there exists a very strong magnetic anisotropy in the compound^2,3,6,14,53–55^, which was firstly ascribed to anisotropic Landé *g* factor^3,55^, and recently to the existence of the off-diagonal Γ interaction^,6,53,60^. We compute the magnetic susceptibilities along two prominent field directions, i.e., ${H}_{[11\bar{2}]}$ and *H*_[111]_, and compare them to experiments in Fig. 2b^14,53,54^. The discussions on different in-plane and tilted fields are left in the Supplementary Note 3.

In Fig. 2b, we show that both the in- and out-of-plane magnetic susceptibilities *χ*_ab_ and ${\chi }_{{\rm{{c}}^{* }}}$ can be well fitted using our *K*-*J*-Γ-${{\Gamma }}^{\prime}$ model, with dominant Kitaev *K*, considerable off-diagonal Γ, as well as similar in-plane (*g*_ab_) and out-of-plane (${g}_{{\rm{{c}}^{* }}}$) Landé factors. Therefore, our many-body simulation results indicate that the anisotropic susceptibilities mainly originate from the off-diagonal Γ coupling (cf. Supplementary Fig. 2), in consistent with the resonant elastic X-ray scattering^6^ and susceptibility measurements^53^. Moreover, with the parameter set of *K*, Γ, ${{\Gamma }}^{\prime}$, *J*, *g*_ab_, and ${g}_{{\rm{{c}}^{* }}}$ determined from our thermodynamics simulations, we compute the magnetization curves $M({H}_{[l,m,n]})=1/N\mathop{\sum }\nolimits_{i = 1}^{N}{g}_{[l,m,n]}{\mu }_{{\rm{B}}}\langle {S}_{i}^{[l,m,n]}\rangle$ along the $[11\bar{2}]$ and [111] directions using DMRG, as shown in Fig. 2c. The two simulated curves, showing clear magnetic anisotropy, are in quantitative agreement with the experimental measurements at very low temperature^3,55^.

### Dynamical spin structure and the M star

The INS measurements on *α*-RuCl_3_ revealed iconic dynamical structure features at low and intermediate energies^14,15^. With the determined *α*-RuCl_3_ model, we compute the dynamical spin structure factors using ED, and compare the results to experiments. First, we show in Fig. 2d the constant **k**-cut at the **k** = Γ and M points (as indicated in Fig. 2e), where a quantitative agreement between theory and experiment can be observed. In particular, the positions of the intensity peak *ω*_Γ_ = 2.69 ± 0.11 meV and *ω*_M_ = 2.2 ± 0.2 meV from the INS measurements^14^, are accurately reproduced with our determined model. For the Γ-point intensity, the double-peak structure, which was observed in experimental measurements^18^, can also be well captured.

We then integrate the INS intensity ${\mathcal{I}}({\bf{k}},\omega )$ over the low- and intermediate-energy regime with the atomic form factor taken into account, and check their **k**-dependence in Fig. 2e-f. In experiment, a structure factor with bright Γ and M points was observed at low energy, and, on the other hand, a renowned six-pointed star shape (dubbed M star^43^) was reported at intermediate energies^14,15^. In Fig. 2e-f, these two characteristic dynamical spin structures are reproduced, in exactly the same energy interval as experiments. Specifically, the zigzag order at low temperature is reflected in the bright M points in the Brillouin zone (BZ) when integrated over [2, 3] meV, and the Γ point in the BZ is also turned on. As the energy interval increases to [4.5, 7.5] meV, the M star emerges as the zigzag correlation is weakened while the continuous dispersion near the Γ point remains prominent. The round Γ peak, which also appears in the pure Kitaev model, is consistent with the strong Kitaev term in our *α*-RuCl_3_ model.

### Suppressing the zigzag order by in-plane fields

**Fig. 4:**
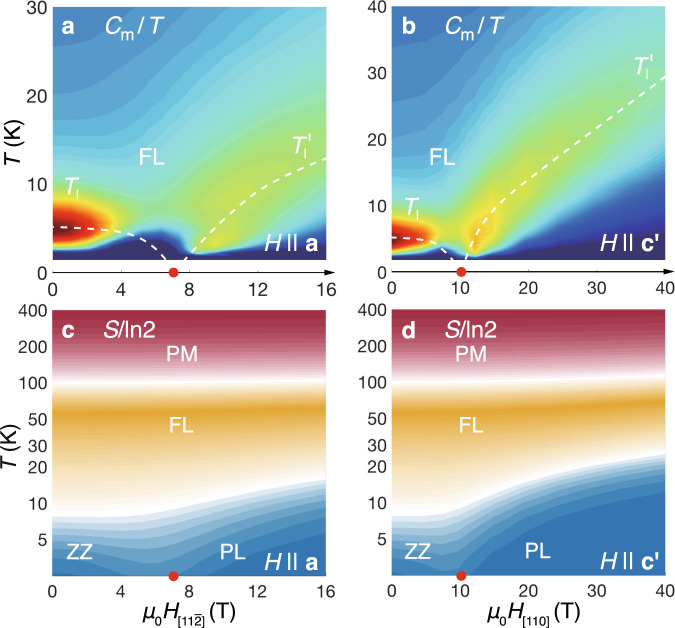
Contour plots of *C*_m_/*T* and the isentropes. The magnetic specific heat *C*_m_/*T* under ${H}_{[11\bar{2}]}\parallel {\bf{a}}$ and ${H}_{[110]}\parallel {\bf{c}}^{\prime}$ are shown in (**a**) and (**b**), respectively. The white dashed lines indicate the low-temperature scale *T*_l_ and $T^{\prime}$ and are guides for the eye. The red dots on the *T* = 0 axis denote the QPT at ${h}_{[11\bar{2}]}^{c}\simeq 7$ T and ${h}_{[110]}^{c}\simeq 10$ T. The isentropes along the two field directions are shown in (**c**) and (**d**), where we find an intermediate regime with fractional thermal entropies $\sim ({\mathrm{ln}}\,2)/2$.

In experiments, the low-*T* zigzag magnetic order has been observed to be suppressed by the in-plane magnetic fields above 7-8 T^3,17,18,55^. We hereby investigate this field-induced effect by computing the spin structure factors under finite fields. The M-point peak of the structure factor *S*(M) in the *T*-${H}_{[11\bar{2}]}$ plane characterizes the zigzag magnetic order as shown in Fig. 3a. The derivatives $\frac{dS({\rm{M}})}{dT}{| }_{H = 0}$ and $\frac{dS({\rm{M}})}{dH}{| }_{T = 1.9{\rm{K}}}$ are calculated in Fig. 3b-c, which can only show a round peak at the transition as limited by our finite-size simulation. For *H* = 0, the turning temperature is at about 7 K, below which the zigzag order builds up; on the other hand, the isothermal *M*-*H* curves in Fig. 3c suggest a transition point at ${h}_{[11\bar{2}]}^{c}={\mu }_{0}{H}_{[11\bar{2}]}\simeq 7$ T, beyond which the zigzag order is suppressed. Correspondingly, in Fig. 4c [and also in Fig. 4d], the low-temperature scale *T*_l_ decreases as the fields increase, initially very slow for small fields and then quickly approaches zero only in the field regime near the critical point, again in very good consistency with experimental measurements^3,17,20^.

Besides, from the contour plots of *C*_m_/*T* and the isentropes in Fig. 4a, c, one can also recognize the critical temperature and field consistent with the above estimations. Moreover, when the field direction is tilted about 55^∘^ away from the **a** axis in the **a**-c^*^ plane (i.e., *H*_[110]_ along the $c^{\prime}$ axis), as shown in Fig. 4b, d, our model calculations suggest a critical field ${h}_{[110]}^{c}={\mu }_{0}{H}_{[110]}\simeq 10$ T with suppressed zigzag order, in accordance with recent NMR probe^20^. Overall, the excellent agreements of the finite-field simulations with different experiments further confirm our *K*-*J*-Γ-${{\Gamma }}^{\prime}$ model as an accurate description of the Kitaev material *α*-RuCl_3_.

### Finite-*T* phase diagram under in-plane fields

Despite intensive experimental and theoretical studies, the phase diagram of *α*-RuCl_3_ under in-plane fields remains an interesting open question. The thermal Hall^29^, Raman scattering^22^, and thermal expansion^61^ measurements suggest the existence of an intermediate QSL phase between the zigzag and polarized phases. On the other hand, the magnetization^3,55^, INS^18^, NMR^17,20,21^, ESR^23^, Grüneisen parameter^62^, and magnetic torque measurements^27^ support a single-transition scenario (leaving aside the transition between two zigzag phases due to different inter-layer stackings^63^). Nevertheless, most experiments found signatures of fractional excitations at finite temperature, although an alternative multi-magnon interpretation has also been proposed^23^.

Now with the accurate *α*-RuCl_3_ model and multiple many-body computation approaches, we aim to determine the phase diagram and nature of the field-driven phase(s). Our main results are summarized in Fig. 1c, e, where a single quantum phase transition (QPT) is observed as the in-plane fields ${H}_{[11\bar{2}]}$ increases. Both VMC and DMRG calculations find a trivial polarized phase in the large-field side (${\mu }_{0}{H}_{[11\bar{2}]}\,> \,{h}_{[11\bar{2}]}^{c}$), as evidenced by the magnetization curve in Fig. 2c as well as the results in Supplementary Note 3.

Despite the QSL phase is absent under in-plane fields, we nevertheless find a Kitaev fractional liquid at finite temperature, whose properties are determined by the fractional excitations of the system. For the pure Kitaev model, it has been established that the itinerant Majorana fermions and *Z*_2_ fluxes each releases half of the entropy at two crossover temperature scales^15,58^. Such an intriguing regime is also found robust in the extended Kitaev model with additional non-Kitaev couplings^59^. Now for the realistic *α*-RuCl_3_ model in Eq. (1), we find again the presence of fractional liquid at intermediate *T*. As shown in Fig. 2b (zero field) and Fig. 3d (finite in-plane fields), the intermediate-*T* Curie-Weiss susceptibility can be clearly observed, with the fitted Curie constant $C^{\prime}$ distinct from the high-*T* paramagnetic constant *C*. This indicates the emergence of a novel correlated paramagnetism—Kitaev paramagnetism—in the material *α*-RuCl_3_. The fractional liquid constitutes an exotic finite-temperature quantum state with disordered fluxes and itinerant Majorana fermions, driven by the strong Kitaev interaction that dominates the intermediate-*T* regime^59^.

In Fig. 4c-d of the isentropes, we find that the Kitaev fractional liquid regime is rather broad under either in-plane (${H}_{[11\bar{2}]}$) or tilted (*H*_[110]_) fields. When the field is beyond the critical value, the fractional liquid regime gradually gets narrowed, from high-*T* scale *T*_h_ down to a new lower temperature scale ${T}_{{\rm{l}}}^{\prime}$, below which the field-induced uniform magnetization builds up (see Supplementary Note 3). From the specific heat and isentropes in Fig. 4, we find in the polarized phase ${T}_{{\rm{l}}}^{\prime}$ increases linearly as field increases, suggesting that such a low-*T* scale can be ascribed to the Zeeman energy. At the intermediate temperature, the thermal entropy is around $({\mathrm{ln}}\,2)/2$ [see Fig. 4c-d], indicating that “one-half” of the spin degree of freedom, mainly associated with the itinerant Majorana fermions, has been gradually frozen below *T*_h_.

Besides, we also compute the spin structure factors ${S}^{\gamma \gamma }({\bf{k}})=1/{N}_{{\rm{Bulk}}}\ {\sum }_{i,j\in {\rm{Bulk}}}{e}^{i{\bf{k}}({{\bf{r}}}_{j}-{{\bf{r}}}_{i})}\langle {S}_{i}^{\gamma }{S}_{j}^{\gamma }\rangle$ under an in-plane field of ${\mu }_{0}{H}_{[11\bar{2}]}=4.2$ T in the fractional liquid regime, where *N*_Bulk_ is the number of bulk sites (with left and right outmost columns skipped), *i*, *j* run over the bulk sites, and *γ* = *x*, *y*, *z*. Except for the bright spots at Γ and M points, there appears stripy background in Fig. 3e-g very similar to that observed in the pure Kitaev model^59^, which reflects the extremely short-range and bond-directional spin correlations there. The stripe rotates as the spin component *γ* switches, because the *γ*-type spin correlations ${\langle {S}_{i}^{\gamma }{S}_{j}^{\gamma }\rangle }_{\gamma }$ are nonzero only on the nearest-neighbor *γ*-type bond. As indicated in the realistic model calculations, we propose such distinct features in *S*^*γγ*^(**k**) can be observed in the material *α*-RuCl_3_ via the polarized neutron diffusive scatterings.

### Signature of Majorana fermions and the Kitaev fractional liquid

It has been highly debated that whether there exists a QSL phase under intermediate in-plane fields. Although more recent experiments favor the single-transition scenario^27,62^, there is indeed signature of fractional Majorana fermions and spin liquid observed in the intermediate-field regime^18,21,22,27,29^. Based on the model simulations, here we show that our finite-*T* phase diagram in Fig. 1 provides a consistent scenario that reconciles these different in-plane field experiments.

For example, large^28,64^ or even half-quantized thermal Hall conductivity was observed at intermediate fields and between 4 and 6 K^30,31,65^. However, it has also been reported that the thermal Hall conductivity vanishes rapidly when the field further varies or the temperature lowers below ~2 K^66^. Therefore, one possible explanation, according to our model calculations, is that the ground state under in-plane fields above 7 T is a trivial polarized phase [see Fig. 1c], while the large thermal Hall conductivity at intermediate fields may originate from the Majorana fermion excitations in the finite-*T* fractional liquid^58^.

In the intermediate-*T* fractional liquid regime, the Kitaev interaction is predominating and the system resembles a pure Kitaev model under external fields and at a finite temperature. This effect is particularly prominent as the field approaches the intermediate regime, i.e., near the quantum critical point, where the fractional liquid can persist to much lower temperature. Matter of fact, given a fixed low temperature, when the field is too small or too large, the system leaves the fractional liquid regime (cf. Fig. 4) and the signatures of the fractional excitation become blurred, as if there were a finite-field window of “intermediate spin liquid phase”. Such fractional liquid constitutes a Majorana metal state with a Fermi surface^58,59^, accounting possibly for the observed quantum oscillation in longitudinal thermal transport^66^. Besides thermal transport, the fractional liquid dominated by fractional excitations can lead to rich experimental ramifications, e.g., the emergent Curie-Weiss susceptibilities in susceptibility measurements [see Fig. 3d] and the stripy spin structure background in the spin-resolved neutron or resonating X-ray scatterings [Fig. 3e-g], which can be employed to probe the finite-*T* fractional liquid in the compound *α*-RuCl_3_.

### Quantum spin liquid induced by out-of-plane fields

**Fig. 5:**
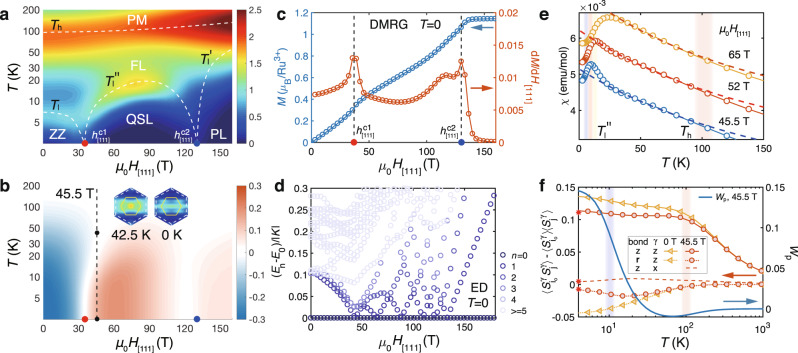
Quantum spin liquid regime of *α*-RuCl_3_ model under the out-of-plane field *H*_[111]_∥ c^*^. **a** Color map of the specific heat *C*_m_, where a double-peak feature appears at both low and intermediate fields. The white dashed lines, with *T*_h_, *T*_l_, and ${T}_{{\rm{l}}}^{^{\prime\prime} }$ determined from the specific heat and ${T}_{{\rm{l}}}^{\prime}$ from spin structure (cf. Supplementary Note 4), represent the phase boundaries and are guides for the eye. **b** shows the *Z*_2_ flux *W*_p_, where the regime with positive(negative) *W*_p_ is plotted in orange(blue). The zero and finite-temperature spin structure factors ${\tilde{S}}^{zz}({\bf{k}})$ (see main text) at *μ*_0_*H*_[111]_ = 45.5 T are shown in the inset of (**b**) and plotted with the same colorbar as that in Fig. 3e-g. **c** Shows the *T* = 0 magnetization curve and its derivative d*M*/d*H*_[111]_, where the two peaks can be clearly identified at *μ*_0_*H*_[111]_ ≃ 35 and 130 T, respectively. The two transition fields, marked by ${h}_{[111]}^{c1}$ and ${h}_{[111]}^{c2}$, are also indicated in panels **a** and **b**. **d** ED Energy spectra *E*_n_-*E*_0_ under *H*_[111]_ fields, with gapless low-energy excitations emphasized by dark colored symbols. **e** The magnetic susceptibility with an intermediate-*T* Curie-Weiss behavior, with the fitted $C^{\prime} \simeq$ 1.22, 1.14, and 1.00 cm^3^ K/mol and $\theta ^{\prime} \simeq$ -236.8, -217.8, and -187.4 K, for fields *μ*_0_*H*_[111]_ = 45.5, 52 and 65 T, respectively. The susceptibility curves in **e** are shifted vertically by a constant 0.001 emu/mol for clarify. **f** shows the temperature dependence of the spin correlations (left *y*-axis) and the flux *W*_p_ (right *y*-axis), where the correlations $\langle {S}_{{i}_{0}}^{z}{S}_{j}^{z}\rangle$- $\langle {S}_{{i}_{0}}^{z}\rangle \langle {S}_{j}^{z}\rangle$ are measured on both *z*- and **r**-bonds [see Fig. 1a], and $\langle {S}_{{i}_{0}}^{x}{S}_{j}^{x}\rangle$- $\langle {S}_{{i}_{0}}^{x}\rangle \langle {S}_{j}^{x}\rangle$ on the *z*-bond, under two different fields of 0 and 45.5 T (with *i*_0_ a fixed central reference site). The low-*T* XTRG data are shown to converge to the *T* = 0 DMRG results, with the latter marked as red asterisks on the left vertical axis. The flux expectation *W*_p_ are measured under *μ*_0_*H*_[111]_ = 45.5 T, which increases rapidly around low-*T* scale ${T}_{{\rm{l}}}^{^{\prime\prime} }$.

**Fig. 6:**
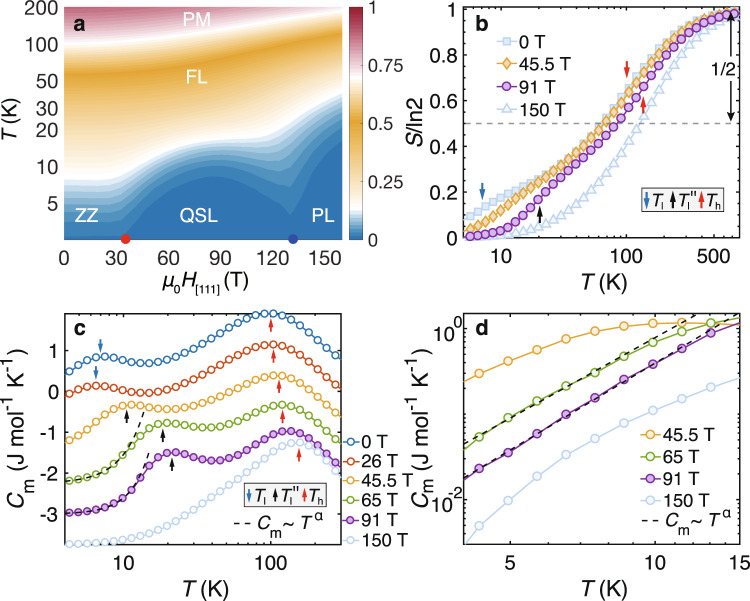
Fractional entropy and specific heat under out-of-plane fields. **a** The contour plot of thermal entropy *S*/ln2, where the two critical fields (red and blue dots) are clearly signaled by the two dips in the isentropic lines. The entropy curves *S*(*T*) are shown in (**b**), where half of the entropy ${{\Delta }}S=({\mathrm{ln}}\,2)/2$ is released around the high temperature scale *T*_h_, while the rest half released either by forming zigzag order (below ${h}_{[111]}^{c1}$, see, e.g., the 0 T curve), or freezing *Z*_2_ flux (between ${h}_{[111]}^{c1}$ and ${h}_{[111]}^{c2}$, e.g., the 45.5 T and 91 T curves), at the lower-temperature scale *T*_l_ and ${T}_{{\rm{l}}}^{^{\prime\prime} }$ denoted by the arrows. For field above ${h}_{[111]}^{c2}$ the system gradually crossover to the polarized states at low temperature. **c** shows the specific heat *C*_m_ curves under various fields, with the blue(black) arrows indicating the low-*T* scales *T*_l_(${T}_{{\rm{l}}}^{^{\prime\prime} }$), and the red arrows for *T*_h_ which increases as the field enhances. The *C*_m_ curves are shifted vertically by a constant 0.75 J mol^−1^ K^−1^ for clarify. In the QSL regime, the low-*T* specific heat show algebraic behaviors in **c**, also zoomed in and plotted with log-log scale in **d**, and we plot a power-law *C*_m_ ~ *T*^*α*^ in both panels as guides for the eye, with *α* estimated to be around 2.8-3.4 based on our YC4 calculations.

Now we apply the *H*_[111]_∥c^*^ field out of the plane and investigate the field-induced quantum phases in *α*-RuCl_3_. As shown in the phase diagram in Fig. 1d, f, under the *H*_[111]_ fields a field-induced QSL phase emerges at intermediate fields between the zigzag and the polarized phases, confirmed in both the thermal and ground-state calculations. The existence of two QPTs and an intermediate phase can also be seen from the color maps of *C*_m_, *Z*_2_ fluxes, and thermal entropies shown in Figs. 5a-b and 6a.

To accurately nail down the two QPTs, we plot the c^*^ DMRG magnetization curve *M*(*H*_[111]_) and the derivative *dM*/*dH*_[111]_ in Fig. 5c, together with the ED energy spectra in Fig. 5d, from which the ground-state phase diagram can be determined (cf. Fig. 1f). In particular, the lower transition field, ${h}_{[111]}^{c1}\simeq 35$ T, estimated from both XTRG and DMRG, is in excellent agreement with recent experiment through measuring the magnetotropic coefficient^27^. The existence of the upper critical field ${h}_{[111]}^{c2}$ at 100 T level can also be probed with current pulsed high field techniques^48^.

Correspondingly, we find in Fig. 5b that the *Z*_2_ flux ${W}_{{\rm{p}}}={2}^{6}\langle {S}_{i}^{x}{S}_{j}^{y}{S}_{k}^{z}{S}_{l}^{x}{S}_{m}^{y}{S}_{n}^{z}\rangle$ (where *i*, *j*, *k*, *l*, *m*, *n* denote the six vertices of a hexagon *p*) changes its sign from negative to positive at ${h}_{[111]}^{c1}$, then to virtually zero at ${h}_{[111]}^{c2}$, and finally converge to very small (positive) values in the polarized phase. These observations of flux signs in different phases are consistent with recent DMRG and tensor network studies on a *K*-Γ-${{\Gamma }}^{\prime}$ model^49,50^, and it is noteworthy that the flux is no longer a strictly conserved quantity as in the pure Kitaev model, the low-*T* expectation values of ∣*W*_p_∣ thus would be very close to 1 only deep in the Kitaev spin liquid phase^49,59,67^.

From the *C*_m_ color map in Fig. 5a and *C*_m_ curves in Fig. 6c, we find double-peaked specific heat curves in the QSL phase, which clearly indicate the two temperature scales, e.g., *T*_h_ ≃ 105 K and ${T}_{{\rm{l}}}^{^{\prime\prime} }\simeq 10$ K for *μ*_0_*H*_[111]_ = 45.5 T. They correspond to the establishment of spin correlations at *T*_h_ and the alignment of *Z*_2_ fluxes at ${T}_{{\rm{l}}}^{^{\prime\prime} }$, respectively, as shown in Fig. 5f. As a result, in Fig. 6a-b the system releases $({\mathrm{ln}}\,2)/2$ thermal entropy around *T*_h_, and the rest half is released at around ${T}_{{\rm{l}}}^{^{\prime\prime} }$. The magnetic susceptibility curves in Fig. 5e fall into an intermediate-*T* Curie-Weiss behavior below *T*_h_ when the spin correlations are established, and deviate such emergent universal behavior when approaching ${T}_{{\rm{l}}}^{^{\prime\prime} }$ as the gauge degree of freedom (flux) gradually freezes.

Furthermore, we study the properties of the QSL phase. We find very peculiar spin correlations as evidenced by the (modified) structure factor

2 $${\tilde{S}}^{_{zz}}({\bf{k}})=\frac{1}{{N}_{{\rm{Bulk}}}}\mathop{\sum}\limits_{i,j\in {\rm{Bulk}}}{e}^{i{\bf{k}}({{\bf{r}}}_{j}-{{\bf{r}}}_{i})}(\langle {S}_{i}^{z}{S}_{j}^{z}\rangle -\langle {S}_{i}^{z}\rangle \langle {S}_{j}^{z}\rangle ).$$

As shown in the inset of Fig. 5b, there appears no prominent peak in ${\tilde{S}}^{_{zz}}({\bf{k}})$ at both finite and zero temperatures, for a typical field of 45.5 T in the QSL phase. As shown in Fig. 5f, we find the dominating nearest-neighboring correlations at *T* > 0 are bond-directional and the longer-range correlations are rather weak, same for the QSL ground state at *T* = 0. This is in sharp contrast to the spin correlations in the zigzag phase. More spin structure results computed with both XTRG and DMRG can be found in Supplementary Note 4. Below the temperature ${T}_{{\rm{l}}}^{^{\prime\prime} }$, we observe an algebraic specific heat behavior as shown in Fig. 6c-d, which strongly suggests a gapless QSL. We remark that the gapless spin liquid in the pure Kitaev model also has bond-directional and extremely short-range spin correlations^13^. The similar features of spin correlations in this QSL state may be owing to the predominant Kitaev interaction in our model.

Overall, under the intermediate fields between ${h}_{[111]}^{c1}$ and ${h}_{[111]}^{c2}$, and below the low-temperature scale ${T}_{{\rm{l}}}^{^{\prime\prime} }$, our model calculations predict the presence of the long-sought QSL phase in the compound *α*-RuCl_3_.

## Discussion

First, we discuss the nature of the QSL driven by the out-of-plane fields. In Fig. 5d, the ED calculation suggests a gapless spectrum in the intermediate phase, and the DMRG simulations on long cylinders find the logarithmic correction in the entanglement entropy scaling (see Supplementary Note 4), which further supports a gapless QSL phase. On the other hand, the VMC calculations identify the intermediate phase as an Abelian chiral spin liquid, which is topologically nontrivial with a quantized Chern number *ν* = 2. Overall, various approaches consistently find the same scenario of two QPTs and an intermediate-field QSL phase under high magnetic fields [cf. Fig. 1d, f]. It is worth noticing that a similar scenario of a gapless QSL phase induced by out-of-plane fields has also been revealed in a Kitaev-Heisenberg model with *K* > 0 and *J* < 0^68^, where the intermediate QSL was found to be smoothly connected to the field-induced spin liquid in a pure AF Kitaev model^67,69,70^. We note the extended AF Kitaev model in Ref. ^68^ can be transformed into a *K*-*J*-Γ-${{\Gamma }}^{\prime}$ model with FM Kitaev term (and other non-Kitaev terms still distinct from our model) through a global spin rotation^71^. Therefore, despite the rather different spin structure factors of the field-induced QSL in the AF Kitaev model from ours, it is interesting to explore the possible connections between the two in the future.

Based on our *α*-RuCl_3_ model and precise many-body calculations, we offer concrete experimental proposals for detecting the intermediate QSL phase via the magnetothermodynamic measurements under high magnetic fields. The two QPTs are within the scope of contemporary technique of pulsed high fields, and can be confirmed by measuring the magnetization curves^47,48^. The specific heat measurements can also be employed to confirm the two-transition scenario and the high-field gapless QSL states. As field increases, the lower temperature scale *T*_l_ first decreases to zero as the zigzag order is suppressed, and then rises up again (i.e., ${T}_{{\rm{l}}}^{^{\prime\prime} }$) in the QSL phase [cf. Fig. 5a]. As the specific heat exhibits a double-peak structure in the high-field QSL regime, the thermal entropy correspondingly undergoes a two-step release in the QSL phase and exhibits a quasi-plateau near the fractional entropy $({\mathrm{ln}}\,2)/2$ [cf. 45.5 T and 91 T lines in Fig. 6b]. This, together with the low-*T* (below ${T}_{{\rm{l}}}^{^{\prime\prime} }$) algebraic specific heat behavior reflecting the gapless excitations, can be probed through high-field calorimetry^46^.

Lastly, it is interesting to note that the emergent high-field QSL under out-of-plane fields may be closely related to the off-diagonal Γ term (see, e.g., Refs. ^49,50^) in the compound *α*-RuCl_3_. The Γ term has relatively small influences in **ab** plane, while introduces strong effects along the c^*^ axis — from which the magnetic anisotropy in *α*-RuCl_3_ mainly originates. The zigzag order can be suppressed by relatively small in-plane fields and the system enters the polarized phase, as the Γ term does not provide a strong “protection” of both zigzag and QSL phases under in-plane fields (recall the QSL phase in pure FM Kitaev model is fragile under external fields^59,67,72^). The situation is very different for out-of-plane fields, where the Kitaev (and also Γ) interactions survive the QSL after the zigzag order is suppressed by high fields. Intuitively, the emergence of this QSL phase can therefore be ascribed to the strong competition between the Γ interaction and magnetic field along the hard axis c^*^ of *α*-RuCl_3_. With such insight, we expect a smaller critical field for compounds with a less significant Γ interaction. In the fast-moving Kitaev materials studies, such compounds with relatively weaker magnetic anisotropy, e.g., the recent Na_2_Co_2_TeO_6_ and Na_2_Co_2_SbO_6_^73–76^, have been found, which may also host QSL induced by out-of-plane fields at lower field strengths.

## Methods

### Exponential tensor renormalization group

The thermodynamic quantities including the specific heat, magnetic susceptibility, *Z*_2_ flux, and the spin correlations can be computed with the exponential tensor renormalization group (XTRG) method^44,45^ on the Y-type cylinders with width *W* = 4 and length up to *L* = 6 (i.e., YC4 × 6 × 2). We retain up to *D* = 800 states in the XTRG calculations, with truncation errors *ϵ* ≲ 2 × 10^−5^, which guarantees a high accuracy of computed thermal data down to the lowest temperature *T* ≃ 1.9 K. Note the truncation errors in XTRG, different from that in DMRG, directly reflects the relative errors in the free energy and other thermodynamics quantities. The low-*T* data are shown to approach the *T* = 0 DMRG results (see Fig. 5f). In the thermodynamics simulations of the *α*-RuCl_3_ model, one needs to cover a rather wide range of temperatures as the high- and low-*T* scales are different by more than one order of magnitude (100 K vs. 7 K in *α*-RuCl_3_ under zero field). In the XTRG cooling procedure, we represent the initial density matrix *ρ*_0_(*τ*) at a very high temperature *T* ≡ 1/*τ* (with *τ* ≪ 1) as a matrix product operator (MPO), and the series of lower temperature density matrices *ρ*_*n*_(2^*n*^*τ*) (*n* ≥ 1) are obtained by successively multiplying and compressing *ρ*_*n*_ = *ρ*_*n*−1_ ⋅ *ρ*_*n*−1_ via the tensor-network techniques. Thus XTRG is very suitable to deal with such Kitaev model problems, as it cools down the system exponentially fast in temperature^59^.

### Density matrix renormalization group

The ground state properties are computed by the density matrix renormalization group (DMRG) method, which can be considered as a variational algorithm based on the matrix product state (MPS) ansatz. We keep up to *D* = 2048 states to reduce the truncation errors *ϵ* ≲ 1 × 10^−8^ with a very good convergence. The simulations are based on the high-performance MPS algorithm library GraceQ/MPS2^77^.

### Variational Monte Carlo

The ground state of *α*-RuCl_3_ model are evaluated by the variational Monte Carlo (VMC) method based on the fermionic spinon representation. The spin operators are written in quadratic forms of fermionic spinons ${S}_{i}^{m}=\frac{1}{2}{C}_{i}^{\dagger }{\sigma }^{m}{C}_{i},m=x,y,z$ under the local constraint ${\hat{N}}_{i}={c}_{i\uparrow }^{\dagger }{c}_{i\uparrow }+{c}_{i\downarrow }^{\dagger }{c}_{i\downarrow }=1$, where ${C}_{i}^{\dagger }=({c}_{i\uparrow }^{\dagger },{c}_{i\downarrow }^{\dagger })$ and *σ*^*m*^ are Pauli matrices. Through this mapping, the spin interactions are expressed in terms of fermionic operators and are further decoupled into a non-interacting mean-field Hamiltonian *H*_mf_(***R***), where ***R*** denotes a set of parameters (see Supplementary Note 5). Then we perform Gutzwiller projection to the mean-field ground state $\left|{{{\Phi }}}_{{\rm{mf}}}({\boldsymbol{R}})\right\rangle$ to enforce the particle number constraint (${\hat{N}}_{i}=1$). The projected states $\left|{{\Psi }}({\boldsymbol{R}})\right\rangle ={P}_{G}\left|{{{\Phi }}}_{{\rm{mf}}}({\boldsymbol{R}})\right\rangle ={\sum }_{\alpha }f(\alpha )\left|\alpha \right\rangle$ (here *α* stands for the Ising bases in the many-body Hilbert space, same for *β* and *γ* below) provide a series of trial wave functions, depending on the specific choice of the mean-field Hamiltonian *H*_mf_(***R***). Owing to the huge size of the many-body Hilbert space, the energy of the trial state $E({\boldsymbol{R}})=\left\langle {{\Psi }}({\boldsymbol{R}})\right|H\left|{{\Psi }}({\boldsymbol{R}})\right\rangle /\langle {{\Psi }}({\boldsymbol{R}})| {{\Psi }}({\boldsymbol{R}})\rangle ={\sum }_{\alpha }\frac{| f(\alpha ){| }^{2}}{{\sum }_{\gamma }| f(\gamma ){| }^{2}}\left(\right.{\sum }_{\beta }\langle \beta | H| \alpha \rangle \frac{f{(\beta )}^{* }}{f{(\alpha )}^{* }}\left)\right.$ is computed using Monte Carlo sampling. The optimal parameters ***R*** are determined by minimizing the energy *E*(***R***). While the VMC calculations are performed on a relatively small size (up to 128 sites), once the optimal parameters are determined we can plot the spinon dispersion of a QSL state by diagonalizing the mean-field Hamiltonian on a larger lattice size, e.g., 120 × 120 unit cells in practice.

### Exact diagonalization

The 24-site exact diagonalization (ED) is employed to compute the zero-temperature dynamical correlations and energy spectra. The clusters with periodic boundary conditions are depicted in the inset of Fig. 2d and the Supplementary Information, and the *α*-RuCl_3_ model under in-plane fields (${H}_{[11\bar{2}]}\parallel {\bf{a}}$ and ${H}_{[1\bar{1}0]}\parallel {\bf{b}}$) as well as out-of-plane fields *H*_[111]_∥c^*^ have been calculated, as shown in Fig. 5 and Supplementary Note 3. Regarding the dynamical results—the neutron scattering intensity—is defined as

3 $$\begin{array}{ll}{\mathcal{I}}({\bf{k}},\omega )\propto &{f}^{2}({\bf{k}})\int dt\mathop{\sum}\limits_{\mu ,\nu }\big({\delta }_{\mu ,\nu }-{k}_{\mu }{k}_{\nu }/{k}^{2}\big)\\ &\times \mathop{\sum}\limits_{i,j}\langle {S}_{i}^{\mu }(t){S}_{j}^{\nu }(0)\rangle {e}^{i{\bf{k}}\cdot ({{\bf{r}}}_{j}-{{\bf{r}}}_{i})}{e}^{-i\omega t},\\ \end{array}$$

where *f*(**k**) is the atomic form factor of Ru^3+^, which can be fitted by an analytical function as reported in Ref. ^78^. ${S}^{\mu \nu }({\bf{k}},\omega )={\sum }_{i,j}\langle {S}_{i}^{\mu }(t){S}_{j}^{\nu }(0)\rangle {e}^{i{\bf{k}}\cdot ({{\bf{r}}}_{j}-{{\bf{r}}}_{i})}{e}^{-i\omega t}$ is the dynamical spin structure factor, which can be expressed by the continued fraction expansion in the tridiagonal basis of the Hamiltonian using Lanczos iterative method. For the diagonal part,

4 $$\begin{array}{ll}{S}^{\mu \mu }({\bf{k}},\omega )&=-\frac{1}{\pi }{\rm{Im}}\big[\left\langle {\psi }_{0}\right|{\hat{S}}_{-{\bf{k}}}^{\mu }\frac{1}{z-\hat{H}}{\hat{S}}_{{\bf{k}}}^{\mu }\left|{\psi }_{0}\right\rangle \big]\\ &\!\!\!\!\!\!\!\!=-\frac{1}{\pi }{\rm{Im}}\Big[\frac{\left\langle {\psi }_{0}\right|{\hat{S}}_{-{\bf{k}}}^{\mu }{\hat{S}}_{{\bf{k}}}^{\mu }\left|{\psi }_{0}\right\rangle }{z-{a}_{0}-\frac{{b}_{1}^{2}}{z-{a}_{1}-\frac{{b}_{2}^{2}}{z-{a}_{2}-\cdot \cdot \cdot }}}\Big],\end{array}$$

where *z* = *ω* + *E*_0_ + *iη*, *E*_0_ is the ground state energy, $\left|{\psi }_{0}\right\rangle$ is the ground state wave function, *η* is the Lorentzian broadening factor (here we take *η* = 0.5 meV in the calculations, i.e., 0.02 times the Kitaev interaction strength ∣*K*∣ = 25 meV), and *a*_*i*_ (*b*_*i*+1_) is the diagonal (sub-diagonal) matrix element of the tridiagonal Hamiltonian. On the other hand, for the off-diagonal part, we define a Hermitian operator ${\hat{S}}_{{\bf{k}}}^{\mu }+{\hat{S}}_{{\bf{k}}}^{\nu }$ to do the continued fraction expansion,

5 $$\begin{array}{ll}{S}^{(\mu +\nu )(\mu +\nu )}({\bf{k}},\omega )\\ &=-\frac{1}{\pi }{\rm{Im}}\left[\left\langle {\psi }_{0}\right|\big({\hat{S}}_{-{\bf{k}}}^{\mu }+{\hat{S}}_{-{\bf{k}}}^{\nu }\big)\frac{1}{z-\hat{H}}\big({\hat{S}}_{{\bf{k}}}^{\mu }+{\hat{S}}_{{\bf{k}}}^{\nu }\big)\left|{\psi }_{0}\right\rangle \right]\\ &\kern-4.3pc=-\frac{1}{\pi }{\rm{Im}}\left[\frac{\left\langle {\psi }_{0}\right|({\hat{S}}_{-{\bf{k}}}^{\mu }+{\hat{S}}_{-{\bf{k}}}^{\nu })({\hat{S}}_{{\bf{k}}}^{\mu }+{\hat{S}}_{{\bf{k}}}^{\nu })\left|{\psi }_{0}\right\rangle }{z-{a}_{0}-\frac{{b}_{1}^{2}}{z-{a}_{1}-\frac{{b}_{2}^{2}}{z-{a}_{2}-\cdot \cdot \cdot }}}\right].\end{array}$$

Then the off-diagonal *S*^*μν*^(**k**, *ω*) can be computed by *S*^*μν*^(**k**, *ω*) + *S*^*νμ*^(**k**, *ω*) = *S*^(*μ*+*ν*) (*μ*+*ν*)^(**k**, *ω*) − *S*^*μμ*^(**k**, *ω*) − *S*^*νν*^(**k**, *ω*). Following the INS experiments^14^, the shown scattering intensities in Fig. 2 are integrated over perpendicular momenta *k*_*z*_ ∈ [ − 5*π*, 5*π*], assuming perfect two-dimensionality of *α*-RuCl_3_ in the ED calculations.

## Supplementary information

Supplementary Information

## Data Availability

The data that support the findings of this study are available from the corresponding author upon reasonable request.

## Code availability

All numerical codes in this paper are available upon request to the authors.
