# Supplementary material for: Identification of magnetic interactions and high-field quantum spin liquid in α-RuCl3
Source: Nat Commun. 2021 Jun 29;12:4007. doi: 10.1038/s41467-021-24257-8 (PMC8242101; doi:10.1038/s41467-021-24257-8)
Supplement: Supplementary file 1 — Supplementary Information [file 41467_2021_24257_MOESM1_ESM.pdf]

Supplementary Information for

Identification of Magnetic Interactions and High-field Quantum Spin Liquid in α-RuCl$_3$

Li *et al.*

Supplementary Note 1. Determination of the Effective α-RuCl$_3$ Hamiltonian

Fitting the model parameters from thermodynamic measurements. In this section we show the workflow of the model parameter fittings. By performing exponential tensor renormalization (XTRG) calculations on a YC4×4×2 lattice, we scan the parameter space spanned by the couplings $[K, \Gamma, \Gamma', J]$, and fit the specific heat $C_{\rm m}(T)$, in-plane ($\chi_{\rm ab}$) and out-of-plane susceptibilities ($\chi_{\rm c^*}$) measured under a small magnetic field $\mu_0 H \simeq 1$ T. From the susceptibility simulations, we also determine the corresponding g-factors, $g_{\rm ab}$ and $g_{\rm c^*}$, along with other couplings. Given the determined parameters, we can compute the static spin structure factors, and confirm the appearance of zigzag magnetic order at low temperature (see Figs. 2,3 in the main text). XTRG and density matrix renormalization group (DMRG) are employed to compute the magnetization curve and compared to experiments directly. Exact diagonalization (ED) calculations are performed on 24-site clusters [see Supplementary Fig. 4(a,b) insets], from which we obtain the dynamical spin structure factors.

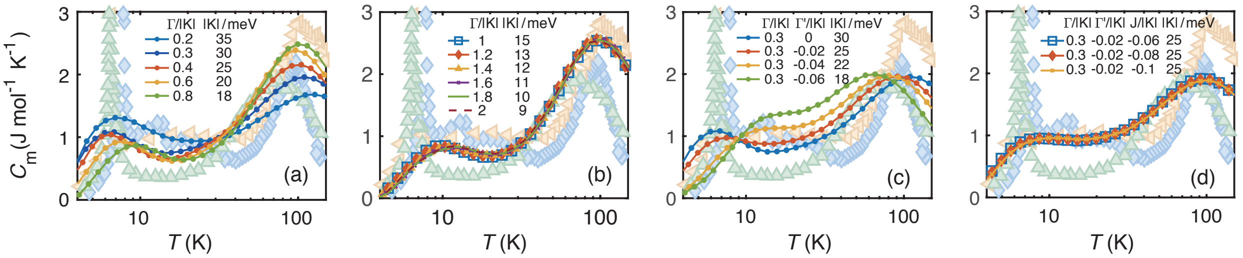

Supplementary Figure 1. **Simulations of the magnetic specific heat $C_{\rm m}$.** (a,b) show the $C_{\rm m}$ curves with different Γ terms, with rest interactions temporarily set to be zero. (c) shows the simulated $C_{\rm m}$ with various Γ' couplings and fixed $\Gamma/|K| = 0.3$, and (d) checks the effects of Heisenberg term J with fixed $\Gamma = 0.3$ and $\Gamma' = -0.02$. All those curves are compared with experimental data [1–3], which are shown in the background. The energy scale $|K|$ is tuned adaptively in different cases to fit the $C_{\rm m}$ curves.

To be concrete, we show in Supplementary Figs. 1, 2 part of our simulated data in the thermodynamic properties. In Supplementary Fig. 1(a,b), we start with scanning over various Γ values with ferromagnetic $K < 0$, by setting $\Gamma' = J = 0$ at first. As shown in Supplementary Fig. 1(a), the $C_{\rm m}$ curves are sensitive to Γ for $\Gamma < 1$, while the curves do not change much for $\Gamma \geq 1$ in Supplementary Fig. 1(b), given that the energy scale $|K|$ is properly tuned. Therefore, to uniquely pinpoint the parameter $\Gamma/|K|$, we need to include more thermodynamic measurements like the magnetic susceptibility.

As shown in Supplementary Fig. 2(a,b), as the Γ interaction increases, the height of computed susceptibility peak decreases accordingly and deviates the experimental in-plane susceptibility $\chi_{\rm ab}$ curves. After a thorough scanning, we find $\Gamma = 0.3$ constitutes an overall optimal parameter in our model fittings of the specific heat and susceptibility. With the given Γ, we find in Supplementary Fig. 2(c) the susceptibility

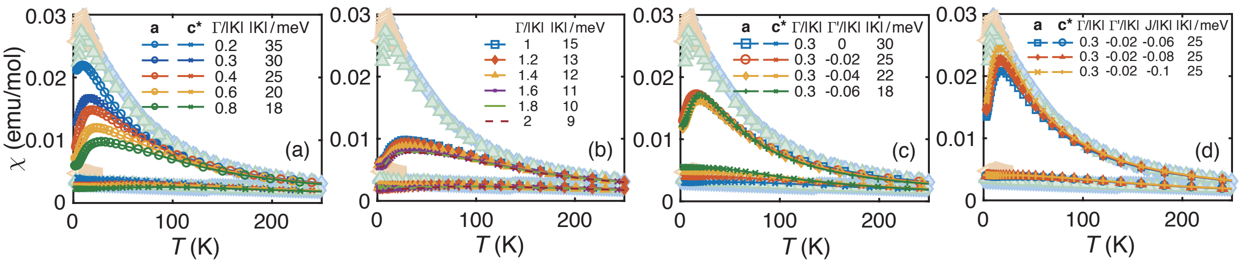

Supplementary Figure 2. **Simulations of the magnetic susceptibility** $\chi_{\rm ab}$ **and** $\chi_{\rm c^*}$**.** (a,b) The susceptibility results, including the in-plane susceptibility $\chi_{\rm ab}$ computed under fields $H_{[11\bar{2}]} \parallel$ **a** and the out-of-plane one $\chi_{\rm c^*}$ under $H_{[111]} \parallel$ **c***, are shown with different $\Gamma/|K|$ values. (c) shows the fittings with various Γ' and a fixed $\Gamma = 0.3$, and (d) the various Heisenberg J with fixed $\Gamma = 0.3$ and $\Gamma' = -0.02$. The two g-factors, i.e., $g_{\rm ab}$ and $g_{\rm c^*}$, are determined to be between 2.2-2.5 (depending on other coupling parameters) from fitting the high-temperature susceptibility. The magnetic field $\mu_0 H$ involved in our fittings are between 0.8-1 T, in consistent with the experimental field of 1 T.

curves turn out to be not very sensitive to the small Γ' interactions, while, on the other hand, the low-T peak of $C_{\rm m}$ moves towards higher temperatures as $|\Gamma'|$ increases in Supplementary Fig. 1(c). This can be understood as the Γ' term is crucial for stabilizing the zigzag order when $K < 0$ and thus has strong influences on the low-T peak. Therefore, we fix $\Gamma' = -0.02$ from the specific heat fittings.

Lastly, we determine the nearest-neighboring Heisenberg term J. In Supplementary Fig. 2(d), we find the height of the susceptibility $\chi_{\rm ab}$ peak enhances and thus approaches the experimental curves as J changes from -0.06 to -0.1, while the specific heat $C_{\rm m}$ is not sensitive to J, as shown in Supplementary Fig. 1(d). From these careful scanings in the parameter space, we determine the parameter set as $[\Gamma/|K|, \Gamma'/|K|, J/|K|, g_{\rm ab}, g_{\rm c^*}] = [0.3, -0.02, -0.1, 2.5, 2.3]$ with $K = -25$ meV, which can very well fit both the specific heat as well as the in- and out-of-plane magnetic susceptibilities.

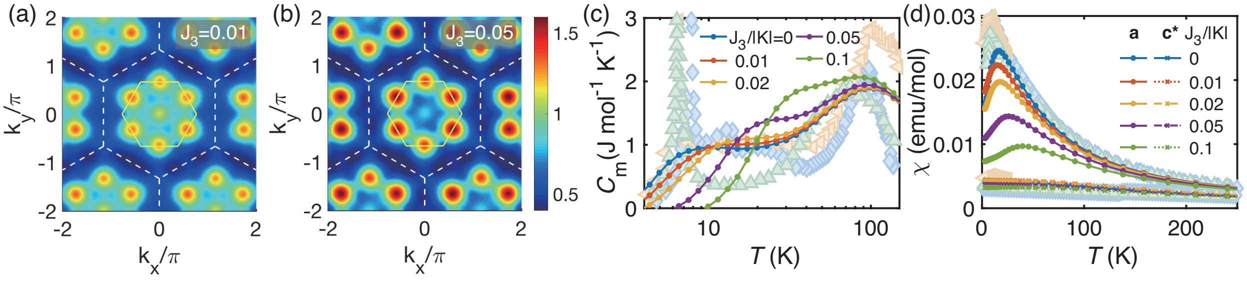

Supplementary Figure 3. **Effects of the** J_3 **coupling in magnetic structure and thermodynamics.** (a,b) show the static spin structure factors of our α-RuCl$_3$ model ($\Gamma/|K| = 0.3$, $\Gamma'/|K| = -0.02$, $J/|K| = -0.1$) with additional $J_3 = 0.01$ and 0.05, respectively. The results are computed at $T \simeq 2.7$ K, and the increased M-point intensity with J_3 shows that the latter enhances the zigzag order. (c) shows the specific heat $C_{\rm m}$ results with various $J_3 > 0$ interactions, as compared to the experimental data in the background. (d) The in- and out-of-plane susceptibility results of our α-RuCl$_3$ model with additional J_3 values.

The third-nearest neighboring J_3 **interaction.** Besides the parameter set considered above, a third

neighboring Heisenberg interaction J_3 has also been suggested to stabilize the zigzag order for $K < 0$ [4–6], which can play a similar role to the off-diagonal Γ' interaction. In order to explore its effects, we introduce an additional J_3 term to our α-$RuCl_3$ model and show the computed results in Supplementary Fig. 3.

In the spin structure factors in Supplementary Fig. 3(a,b), we find the M-point intensity clearly enhances when J_3 increases from 0.01 to 0.05. At the same time, even a small J_3 can have a considerable impact on the low-temperature thermodynamics and makes our model fittings deviate from the experimental data. In Supplementary Fig. 3(c,d), we find a $J_3 \geq 0.05$ clearly spoils the fittings to C_m and χ: The low-T specific heat peak moves towards higher temperature, and the height of the susceptibility peak gets reduced when increasing J_3. In addition, from Supplementary Fig. 3(b), $J_3 = 0.05$ interaction evidently cripples the spin intensity at the Γ point of the Brillouin zone (BZ). Overall, we find J_3 indeed plays a very similar role as Γ' in stabilizing the zigzag order, and, such a term, if exists, should have a small coupling strength. We therefore leave out J_3 in the main text as well as the discussion below, and mostly focus on the minimal K-J-Γ-Γ' effective model in the main text.

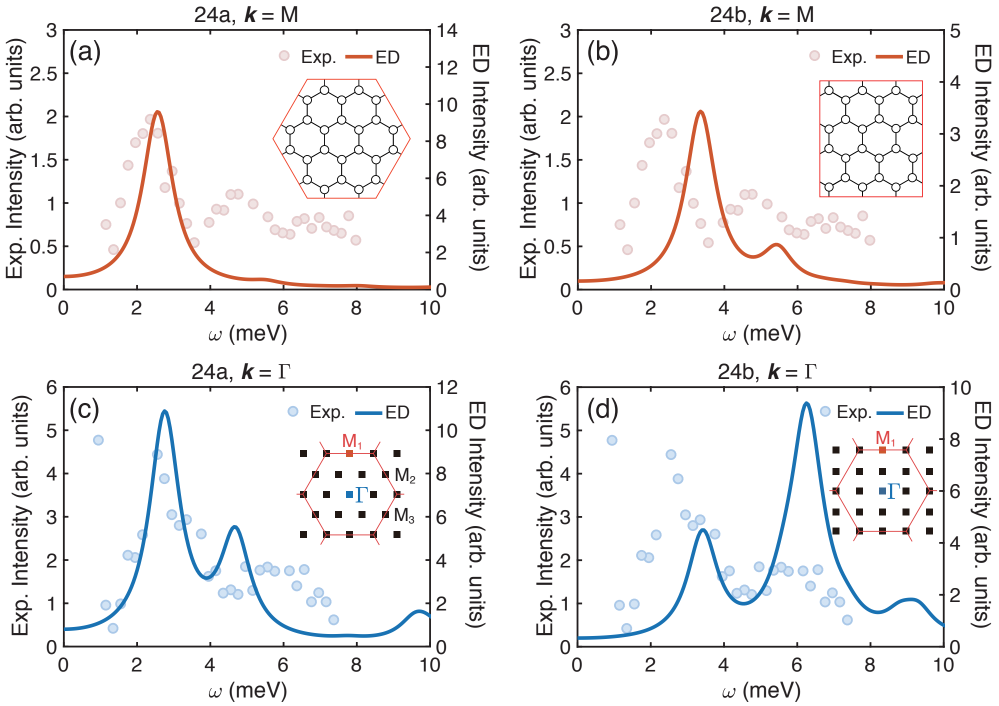

Supplementary Figure 4. **The dynamical ED results of intensity $\mathcal{I}(\mathbf{k}, \omega)$ at the K and Γ points.** (a,b) shows the constant **k**-cut of $\mathcal{I}(\mathbf{k}, \omega)$ at **k** = M and (c,d) at **k** = Γ points. The dynamical data in (a,c) are computed on cluster 24a with its real-space and reciprocal lattices illustrated in panels (a) and (c), respectively. The corresponding results of 24b cluster are shown in (b,d), note there are no M_2 and M_3 points in the BZ of 24b cluster as illustrated in the inset of (d). The intensity peak locations ω_M and ω_Γ are stable [ω_M = 2.5 and 3.3 meV in (a,b), and ω_Γ = 2.7 and 3.4 meV in (c,d), respectively] while the relative intensities between two peaks are found more sensitive to the specific geometries due to the finite-size effects.

Exact diagonalization results of dynamical spin structure factors. In the dynamical simulations, the 24-site cluster with C_3 symmetry (denoted as 24a henceforth) has been widely adopted in ED calculations of the Kitaev model (see, e.g., Refs. [5, 7–9]). Amongst other geometries that are accessible by ED, the 24a cluster is unique as it contains all the high-symmetry points in the BZ [c.f., inset in Supplementary Fig. 4(c)], which is important for dynamical property calculations. Therefore, we choose the 24a cluster in presenting our dynamical data in the main text. Nevertheless, in Supplementary Fig. 4 we also perform dynamical ED simulations on a different 24-site geometry (24b) and compare the results to those of the 24a cluster. It can be seen in Supplementary Fig. 4 that the positions ω_{M} and ω_{Γ} of the intensity peaks as well as the double-peak structure of Γ-intensity curves are qualitatively consistent. Note the 24b cluster shown in Supplementary Fig. 4(b,d) are not C_3 symmetric and thus does not possess all high symmetry moment points.

Magnetic anisotropy and the off-diagonal Γ term. The off-diagonal Γ interaction has been suggested to be responsible for the strong magnetic anisotropy between in- and out-of-plane directions [10, 11]. In a pure Kitaev model without the Γ term, it has been shown that the in-plane (χ_{ab}) and out-of-plane ($\chi_{\mathrm{c^*}}$) susceptibilities are of similar magnitudes [12]. On the other hand, as shown in Supplementary Fig. 2, when the Γ term is introduced, we find the two susceptibility curves are clearly separated, suggesting that the off-diagonal Γ interaction indeed constitutes a resource of strong easy-plane anisotropy observed in α-RuCl$_3$.

Automatic searching of the Hamiltonian parameters. Through the fitting process described above, we have manually determined an accurate set of model parameters of α-RuCl$_3$. As a double-check, below we exploit the recently developed automatic parameter searching technique based on the Bayesian approach [13] to conduct a global optimization of the model parameters in Supplementary Fig. 5. The target is to minimize the fitting loss of the simulated results to measured thermodynamic quantities, including the specific heat C_{m} as well as the in- (χ_{ab}) and out-of-plane ($\chi_{\mathrm{c^*}}$) susceptibilities. Since the specific heat data vary greatly in various measurements and the lower-T peak diverges likely due to 3D effects, we take the mean values of several measurements as the specific heat data $C_{\mathrm{m}}^{\mathrm{exp}}(T)$ used in the fittings. In practice, the loss function $\mathcal{L}$ is designed as follows:

$$\begin{aligned}\mathcal{L} = \log\{\frac{\lambda_\chi}{N_{\chi_{\mathrm{ab}}}} \sum_{T>T_{\mathrm{cut}}} [\chi_{\mathrm{ab}}^{\mathrm{exp}}(T) - \chi_{\mathrm{ab}}^{\mathrm{sim}}(T)]^2 + \frac{\lambda_\chi}{N_{\chi_{\mathrm{c^*}}}} \sum_{T>T_{\mathrm{cut}}} [\chi_{\mathrm{c^*}}^{\mathrm{exp}}(T) - \chi_{\mathrm{c^*}}^{\mathrm{sim}}(T)]^2 \\ + \frac{\mathcal{P}\,\lambda_C}{N_{C_{\mathrm{m}}}} \sum_{T>T'_{\mathrm{cut}}} [C_{\mathrm{m}}^{\mathrm{exp}}(T) - C_{\mathrm{m}}^{\mathrm{sim}}(T)]^2\},\end{aligned} \tag{S1}$$

where $\lambda_C = 1/\max(C_{\mathrm{m}}^{\mathrm{exp}})^2$ and $\lambda_\chi = 1/\max(\chi_{ab}^{\mathrm{exp}})^2$ controls the weights of specific heat and magnetic susceptibility in defining the fitting loss function. $N_{\chi_{\mathrm{ab}}}$, $N_{\chi_{\mathrm{c^*}}}$, and $N_{C_{\mathrm{m}}}$ are the data point numbers in three

experimental curves, respectively. To focus on the fittings of the locations of two specific heat peaks at 100 K and 8 K, respectively, we further introduce a penalty factor $\mathcal{P}$ defined as

$$\mathcal{P} = \begin{cases} \min[\frac{1}{2}\sum_i^{1,2}\exp(\frac{|T_i^{\exp}-T_i^{\rm sim}|}{0.15\,T_i^{\exp}})-1, a] + \min[\frac{1}{2}\sum_i^{1,2}\exp(\frac{|C_i^{\exp}-C_i^{\rm sim}|}{0.25\,C_i^{\exp}})-1, a] & , \text{ two peaks} \\ 2a & , \text{ otherwise} \end{cases} \tag{S2}$$

where $\{T_1, T_2\} = \{100\text{ K}, 8\text{ K}\}$ are the position of the peaks, with $\{C_1, C_2\}$ the specific heat value at corresponding higher and lower T. As our many-body calculations are performed on a finite-size system, we need to introduce the $T_{\rm cut}(T'_{\rm cut})$ as the lowest temperature involved in the fittings, and in practice it is set as $T_{\rm cut} = 25$ K for magnetic susceptibility and $T'_{\rm cut} = 3$ K for the specific heat data. The factor $\mathcal{P}$ emphasizes the double-peak structure as well as the locations and heights of each peaks in the simulated $C_{\rm m}$, where we set an empirical factor $a = 2$ to enlarge the loss function as a penalty for the simulated specific heat curves without double-peak structure.

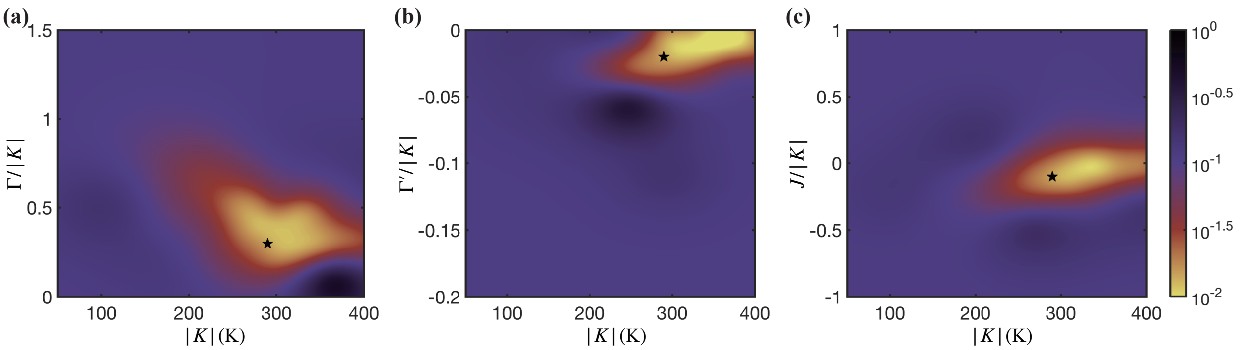

Supplementary Figure 5. The estimated fitting loss $\mathcal{L}$ shown in (a) K-Γ, (b) K-Γ′, and (c) K-J planes. The results are obtained by Bayesian optimization through 120 iterations of XTRG calculations on the YC4×4×2 cylinder. The asterisk represents the optimal parameter set found to also reproduce other major experimental observations including the magnetization curve and dynamical spin structures, which is located within the bright regime with small fitting loss of thermodynamics. The two Landé factors $g_{\rm ab}$ and $g_{\rm c^*}$ at each parameter point are tuned to their optimal values (between 1.5 and 3) to minimize the loss $\mathcal{L}$. Note, when plotting the cross sectors, like the K-Γ plane in (a), the other model parameters are fixed at their optimal values.

In Supplementary Fig. 5, we show the estimated loss $\mathcal{L}$ in the K-Γ, K-Γ′, and K-J planes, respectively. From the color map of $\mathcal{L}$, we see clearly optimal (bright) regimes, and the determined optimal parameter set in the main text is indicated by the asterisk, i.e., $K = -25$ meV, $\Gamma = 0.3\ |K|$, $\Gamma' = -0.02\ |K|$, and $J = -0.1\ |K|$, with the in- and out-of-plane Landé factors found to be $g_{\rm ab} = 2.5$ and $g_{\rm c^*} = 2.3$, respectively, which can fit excellently both the thermodynamic and dynamic measurements in Fig. 2 of the main text.

Supplementary Note 2. Revisit of Various α-RuCl$_3$ Candidate Models

We now employ XTRG to revisit some of the previously proposed α-RuCl$_3$ candidate models in the literature [4, 14–21], where the Kitaev coupling K, Heisenberg J (nearest neighboring), J_3 (third-nearest neighboring), as well as off-diagonal Γ and Γ' terms are considered. We calculated the thermodynamics and static spin structure factors, the main results are summarized in Supplementary Table. 1 and the detailed thermodynamic results are shown in Supplementary Figs. 6 and 7.

Refs.	K (meV)	$\Gamma/\lvert K\rvert$	$\Gamma'/\lvert K\rvert$	$J/\lvert K\rvert$	$J_3/\lvert K\rvert$	$C_{\rm m}$ †	χ_a	χ_{c^*}	zigzag order‡	M-star *
Winter2016 [14]	-6.7	0.985	-0.134	-0.254	0.403	✗	✗	✓	✓	✗
Winter2017 [4]	-5	0.5	/	-0.1	0.1	✗	✗	✗	✓	✗
Wu2018 [15]	-2.8	0.857	/	-0.125	0.121	✗	✗	✗	✓	✗
Cookmeyer2018 [16]	-5	0.5	/	-0.1	0.023	✗	✗	✗	✓	✓
Kim2016 [17]	-6.55	0.802	-0.145	-0.234	/	✗	✓	✓	✓	✗
Suzuki2019 [18]	-24.4	0.215	-0.039	-0.063	/	✓	✓	✓	✓	✗
Ran2017 [19]	-6.8	1.397	/	/	/	✓	✓	✓	✗	✓
Wang2017 [20]	-10.9	0.56	/	-0.028	0.003	✗	✓	✓	✗	✗
Ozel2019 [21]	-3.5	0.671	/	0.131	/	✗	✗	✗	✗	✗
Our model⋆	-25	0.3	-0.02	-0.1	/	✓	✓	✓	✓	✓

† Check if the double-peak feature exists in the calculated $C_{\rm m}$ curves.

‡ Check if the model exhibits a low-T zigzag order.

∗ Check if the dynamical M-star structure exists (taken from Ref. [9], except for our model in the last row).

⋆ Our α-RuCl$_3$ model proposed in the main text.

Supplementary Table 1. **Checklist of various candidate models of α-RuCl$_3$ on typical experimental features.** The "✓ " symbol indicates a good fitting between model calculations and experimental measurements, while "✗ " represents a disagreement between the two.

Specific heat and susceptibility curves. In Supplementary Fig. 6 we show the simulated magnetic specific heat $C_{\rm m}$ and susceptibility χ of various candidate models, and compare them to the experimental measurements. From Supplementary Fig. 6(a-c), we find only the models Suzuki2019 [18] and Ran2017 [19] exhibit a double-peaked $C_{\rm m}$ curve, each located at the characteristic temperature precisely as in experiments. For the rest of revisited candidate models, however, we did not observe the desired double-peak feature in the right temperature window.

The magnetic susceptibility results of the candidate models are shown in Supplementary Fig. 6(d-f), where we find four models out of them, i.e., Kim2016 [17], Suzuki2019 [18], Ran2017 [19], and Wang2017 [20] offer adequate fittings to both in- and out-of-plane susceptibility measurements, with similar Landé factors $g_{\rm ab}$ and g_{c^*} ranging between 2.1 and 2.3 (depending on the specific candidate model).

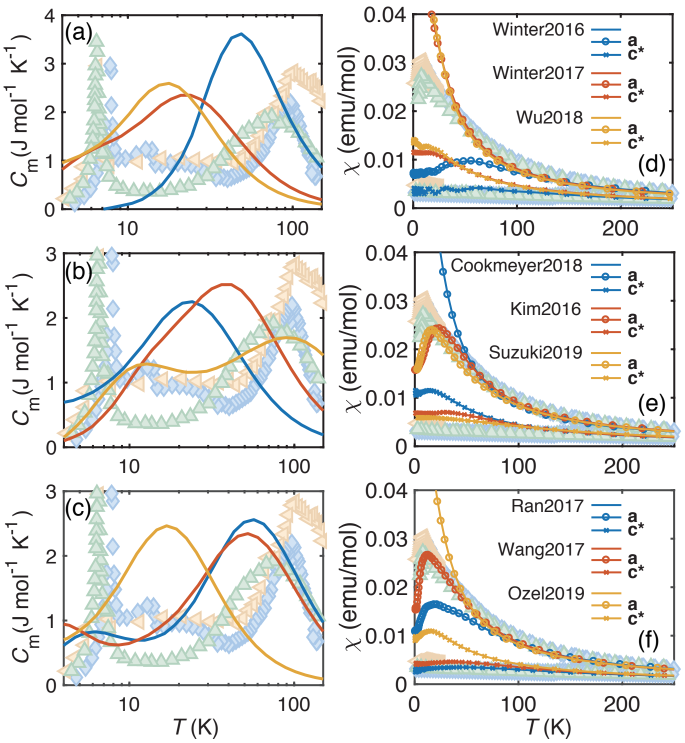

Supplementary Figure 6. Thermodynamic properties of various α-RuCl$_3$ candidate models. (a-c) The magnetic specific heat C_m curves (marked as solid lines) compared to the experimental measurements. (d-f) The susceptibility data with fields $\mu_0 H \parallel$ **a** and **c*** around 1 T. The calculations are performed on YC4×4×2 lattice.

Spin structures and the low-T zigzag order. The compound α-RuCl$_3$ exhibits a zigzag antiferromagnetic order below $T \simeq 7$ K [1–3, 22, 23], which corresponds to a spin structure peak at the M point. Therefore, in Supplementary Fig. 7 we show the static spin structure factors $S(\mathbf{k}) = \sum_{\gamma=\{x,y,z\}} S^{\gamma\gamma}(\mathbf{k})$ at an intermediate $T \sim 40$ K [see Supplementary Fig. 7(a-j)] and a low temperature $T \sim 5$ K [Supplementary Fig. 7(a′-j′)]. Besides the prominent M-peak at low T, we also anticipate a M-star static structure factor emerging at intermediate temperatures, which reflects the short-range spin correlations at both the Γ and M points of the BZ. All these features can be well reproduced in the candidate models Winter2017 [4], Wu2018 [15], Cookmeyer2018 [16], Kim2016 [17], and Suzuki2019 [18]. As for the dynamical structure factors, it has been discussed before in Ref. [9] that only the models Cookmeyer2018 [16] and Ran2017 [19] could reproduce the M-star shape of the inelastic neutron scattering intensity, when integrated over [4.5, 7.5] meV [24].

Overall, as concluded in Ref. [9], we also find no single candidate model revisited here can satisfactorily explain all observed phenomena of experiments on α-RuCl$_3$. For example, both Cookmeyer2018 [16] and Ran2017 [19] have M-star shape in the intermediate-energy dynamical spin structure, however, the former does not fit the specific heat and susceptibility measurements well and the latter does not host a zigzag

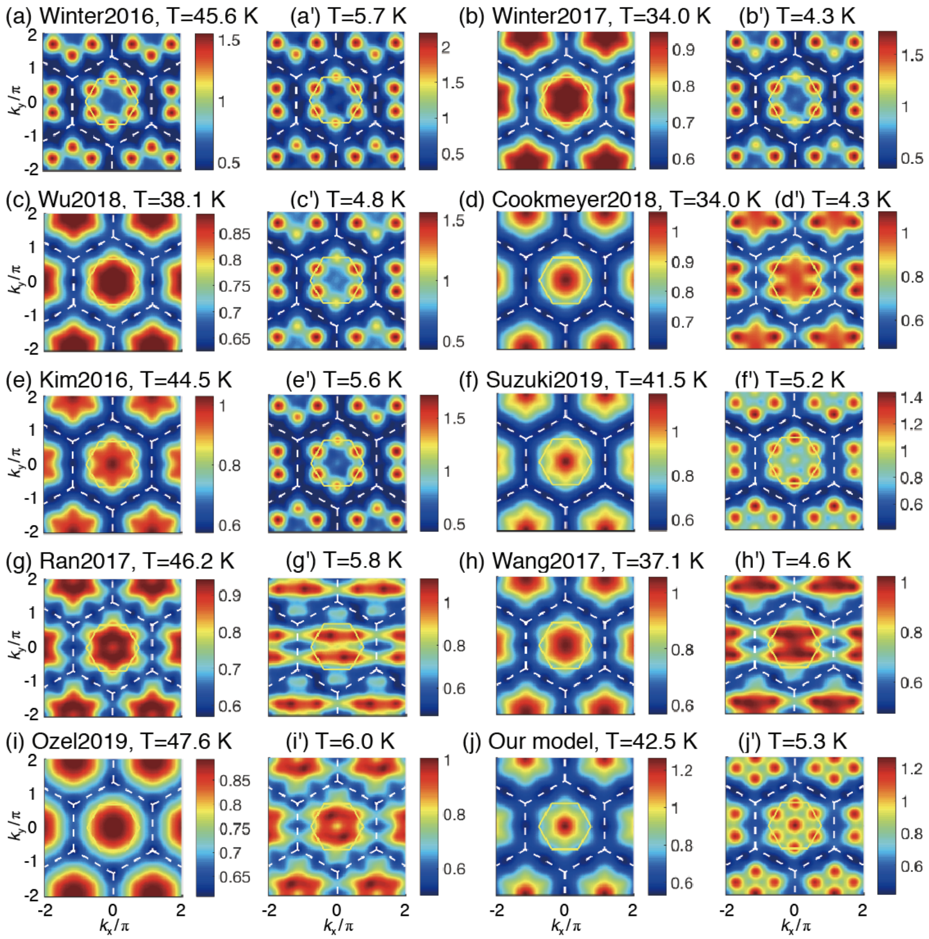

Supplementary Figure 7. **Color maps of the static spin structure factors $S(\mathbf{k})$ of various candidate α-RuCl$_3$ models.** (a-j) show the intermediate-T results and (a$'$-j$'$) show their low-T counterparts.

oder at low temperature. Nevertheless, we note that the model Suzuki2019 [18] can reproduce the prominent thermodynamic features such as the double-peaked specific heat, highly anisotropic susceptibility, and zigzag static spin structures (although the dynamical M star was not found in this candidate model according to Ref. [9]). Our model, on the other hand, accurately describes the spin interactions and explain thus major experimental findings in the compound α-RuCl$_3$.

Supplementary Note 3. α-RuCl$_3$ Model Simulations under In-plane and Tilted Magnetic Fields

In this Note, we show various thermodynamic properties of α-RuCl$_3$ under in-plane and tilted $\mathbf{c}'$ fields (see Fig. 1b in the main text), and compare the simulated results to experimental measurements.

Magnetization curves along the a- and c$'$-axis. In Supplementary Fig. 8(a,b), we show the magnetization curves obtained from the $T = 0$ DMRG calculations on YC4×4×2 geometry. QPT takes place at 7 T and 10 T, for $H_{[11\bar{2}]} \parallel \mathbf{a}$ and $H_{[110]} \parallel \mathbf{c}'$, respectively. The QPT is clearly signaled by the divergent

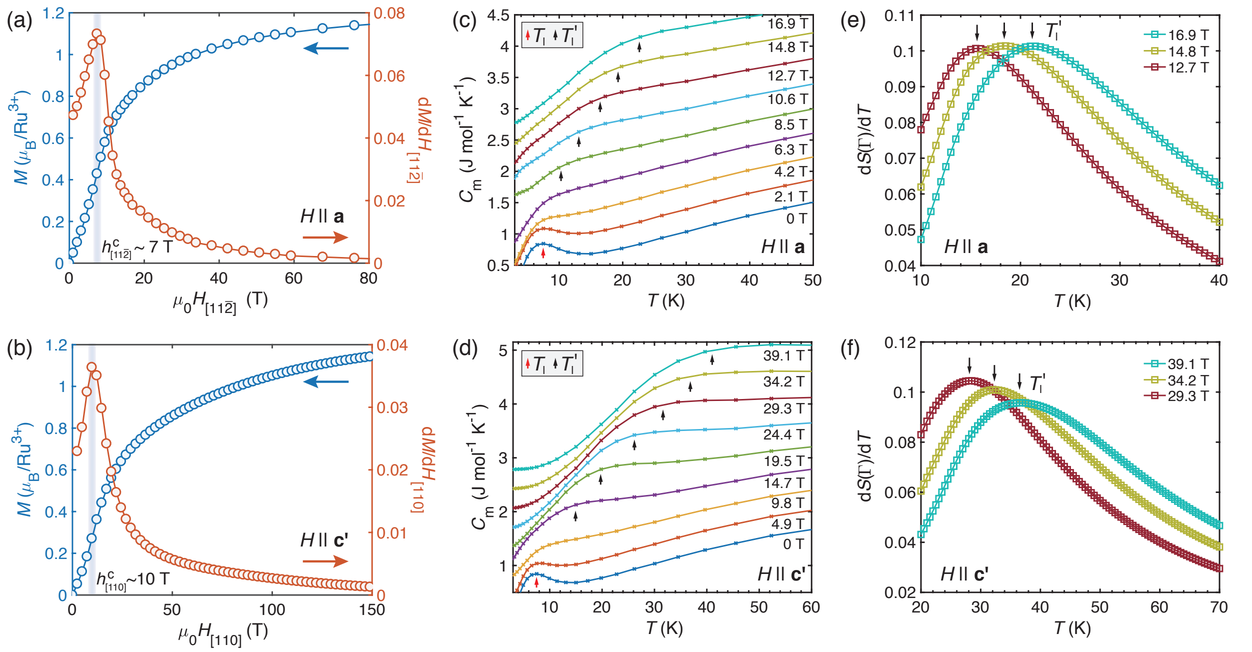

Supplementary Figure 8. **Magnetization curves and thermodynamic properties under fields $H_{[11\bar{2}]} \parallel$ a and $H_{[110]} \parallel$ c′.** The ground-state magnetization curves, under (a) in-plane field $H_{[11\bar{2}]}$ along **a**- and (b) the tilted $H_{[110]}$ along **c**′-axis, are plotted with their derivatives dM/dH, where only one QPT can be observed in both cases. (c, d) Low-temperature specific heat curves $C_{\rm m}$, with each curve shifted by 0.35 J mol^{-1} K^{-1} vertically for clarify. The low-T scale $T_{\rm l}$ and $T_{\rm l}'$ are indicated by the red and black arrows, respectively. (e,f) show the temperature derivatives of the uniform magnetization, i.e., $dS(\Gamma)/dT$, with the peaks located at $T_{\rm l}'$.

derivative dM/dH, where the zigzag order becomes suppressed and the system enters the polarized phase. As the field further increases, the uniform magnetization M gradually approaches the saturation value.

Specific heat under magetic fields and the Zeeman energy scale $T_{\rm l}'$. In Supplementary Fig. 8(c,d), we show the low-T part of the magnetic specific heat curves on YC4×6×2 systems calculated by XTRG. For $\mu_0 H_{[11\bar{2}]} < 7$ T, we find the height of the $C_{\rm m}$ peaks is suppressed by fields, and the low-temperature scale $T_{\rm l}$ associated with the zigzag order, as indicated by the red arrow in Supplementary Fig. 8(c), decreases to zero when the field approaches the critical value, in agreement with experiments [1]. Above that, a new low-temperature scale $T_{\rm l}'$ builds up and moves towards higher temperatures almost linearly vs $H_{[11\bar{2}]}$, as indicated by the black arrows in Supplementary Fig. 8(c). We find $T_{\rm l}'$ is intimately related to the uniform magnetization, as observed from the temperature variation of spin structure intensity $S(\Gamma)$. In Supplementary Fig. 8(e), we show the derivative $\mathrm{d}S(\Gamma)/\mathrm{d}T$ exhibits clear peaks, whose locations are in agreement with the temperature scale $T_{\rm l}'$ determined from the low-temperature peak of $C_{\rm m}$ in Supplementary Fig. 8(c). The peaks moving towards higher temperature as the magnetic field increases, and we thus relate $T_{\rm l}'$ to the Zeeman energy scale. Beside in-plane fields, as depicted in Supplementary Fig. 8(d,f), the

case of $H_{[110]} \parallel \mathbf{c}'$ exhibits very similar behaviors.

Energy spectra under in-plane fields. In Supplementary Fig. 9 we show ED results of the energy spectra on the 24-site cluster (24a), under two in-plane fields $H_{[11\bar{2}]} \parallel \mathbf{a}$ and $H_{[1\bar{1}0]} \parallel \mathbf{b}$ axis [c.f., Supplementary Fig. 9(a)]. The energy spectra results under various fields are plotted in Supplementary Fig. 9(b) and (c), for $H \parallel \mathbf{a}$ and $\mathbf{b}$, respectively. From there we observe the spin gap $(E_1 - E_0)$ changes its behavior at an intermediate field, which can be estimated as the transition field $h^c_{[11\bar{2}]}$ and $h^c_{[1\bar{1}0]}$, and the spectra appear differently along **a** and **b** directions, consistent with the observation in recent experiments [10, 25].

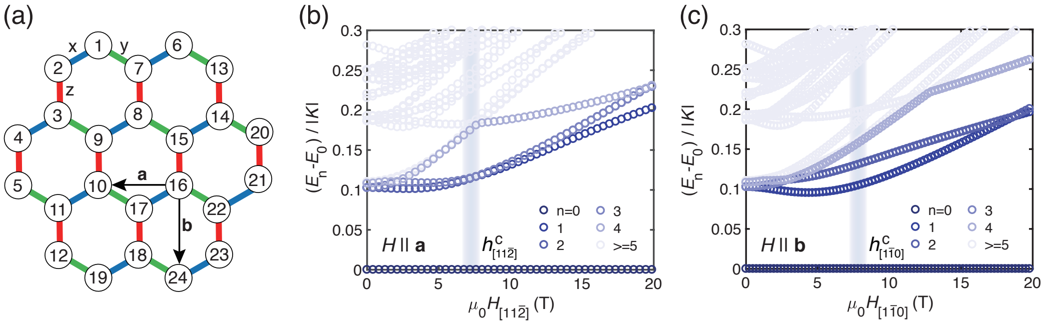

Supplementary Figure 9. **The energy spectra under in-plane magnetic fields.** (a) The 24-site cluster involved in the ED calculations, with the directions of two in-plane fields $H_{[11\bar{2}]} \parallel \mathbf{a}$ and $H_{[1\bar{1}0]} \parallel \mathbf{b}$ indicated by the black arrows. (b,c) show the energy spectra $(E_\mathrm{n} - E_0)$ under the in-plane fields, with the ground-state energy E_0 subtracted. In two cases, the spin gap starts to increase rapidly at about $h^c_{[11\bar{2}]} \simeq 7$ T and $h^c_{[1\bar{1}0]} \simeq 8$ T, respectively, as indicated by the thick vertical lines.

Supplementary Note 4. Quantum Spin Liquid under Out-of-plane Magnetic Fields

Finite-temperature Spin structures under out-of-plane fields. In Supplementary Fig. 10, we plot the spin structure factors at two typical momentum points in the BZ, i.e., $\mathbf{k}$ =M and Γ. From the $S(\mathrm{M})$ curves in Supplementary Fig. 10(a), we find the zigzag order [cf. 13 T and 26 T lines in Supplementary Fig. 10(a)] gets suppressed in the quantum spin liquid (QSL) phase under strong out-of-plane magnetic fields [see, e.g., 45.5 T, 78 T, and 91 T lines in Supplementary Fig. 10(a)]. In the QSL phase, there exists enhanced M-point spin correlation near the emergent lower temperature scale T_1'', as indicated by the black arrows in Supplementary Fig. 10(a). In Supplementary Fig. 10(b), we show the temperature derivative $-dS(\Gamma)/dT$, whose peak position signals the low-temperature scale T_1', below which the field-induced uniform magnetization gets established, i.e., T_1' is a crossover temperature scale to the polarized state. As shown in Supplementary Fig. 10(b), T_1' moves to higher temperatures as the field increases.

In the main text, we have discussed the static spin-structure factors $\tilde{S}^{zz}(\mathbf{k})$ under an out-of-plane field of 45.5 T (cf. the insets of Fig. 5b of the main text). In Supplementary Fig. 10(c,d), we supplement with

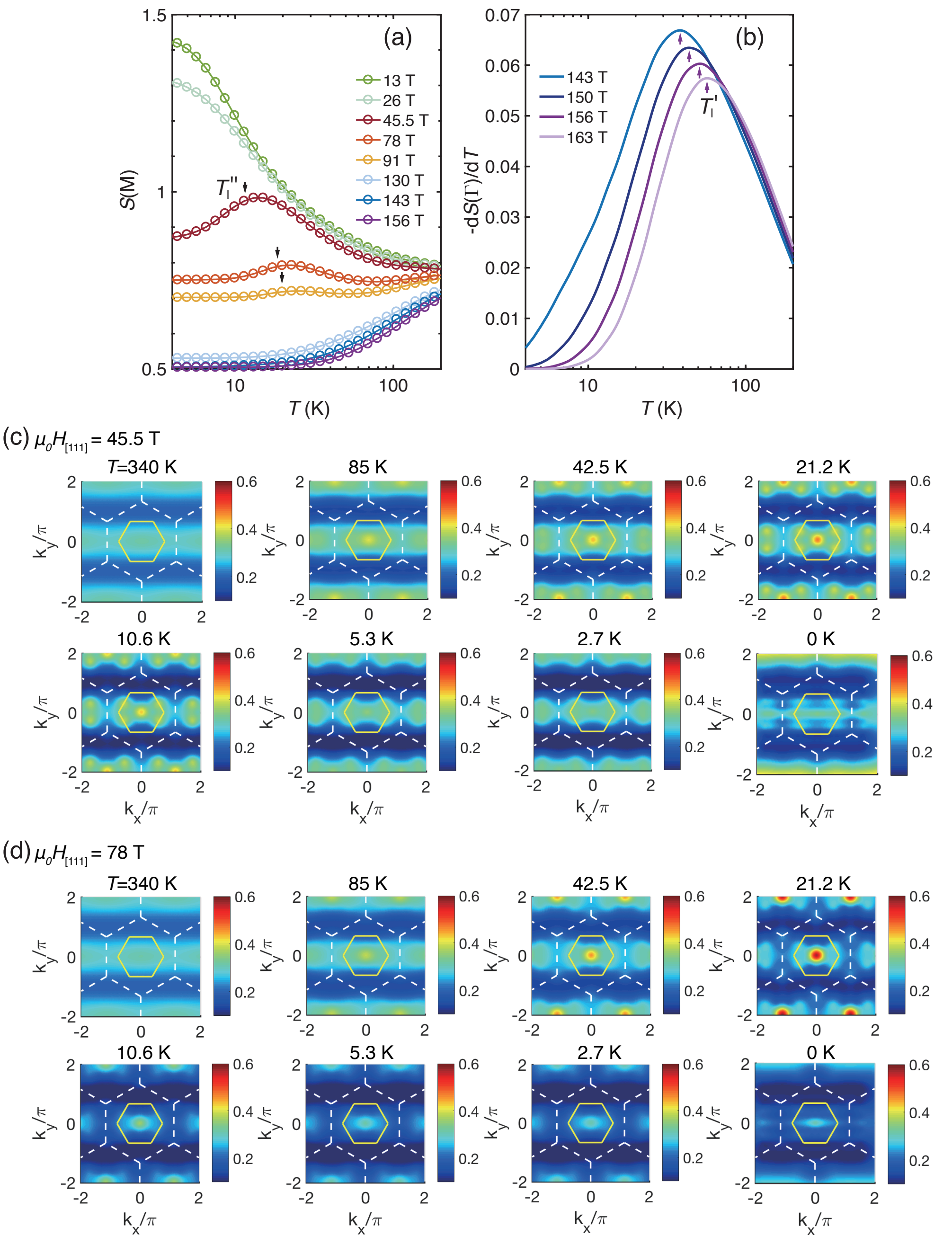

Supplementary Figure 10. **The spin structure factors under out-of-plane $H_{[111]}$ fields.** We show (a) the $S(\mathrm{M})$ and (b) $-dS(\Gamma)/dT$ curves with temperature T, which are computed on the YC4×6×2 lattice. The black arrows in (a) represent the temperature scale T_l'' determined from the low-T peak of the specific heat in Fig. 6 of the main text, and the purple ones in (b) indicates the low-T scales T_l' which locates at the peak of $-dS(\Gamma)/dT$. The static spin-structure factors $\tilde{S}^{zz}(\mathbf{k})$ [following the definition in Eq. (2) of the main text] under the field of $\mu_0 H_{[111]} = 45.5$ T and 78 T are shown in (c) and (d), respectively, from high to low temperatures and compared to the ground-state results. The data are plotted here in the same colorbar as that in Fig. 3e-g of the main text.

XTRG results of $\tilde{S}^{zz}(\mathbf{k})$ at various temperatures and under two fields, 45.5 T and 78 T. For each case, we compare the finite-T XTRG results to the $T = 0$ data obtained by DMRG. At high temperature, i.e., $T = 340$ K, the spin structure is virtually featureless for both cases, with only a faint stripe due to the strong

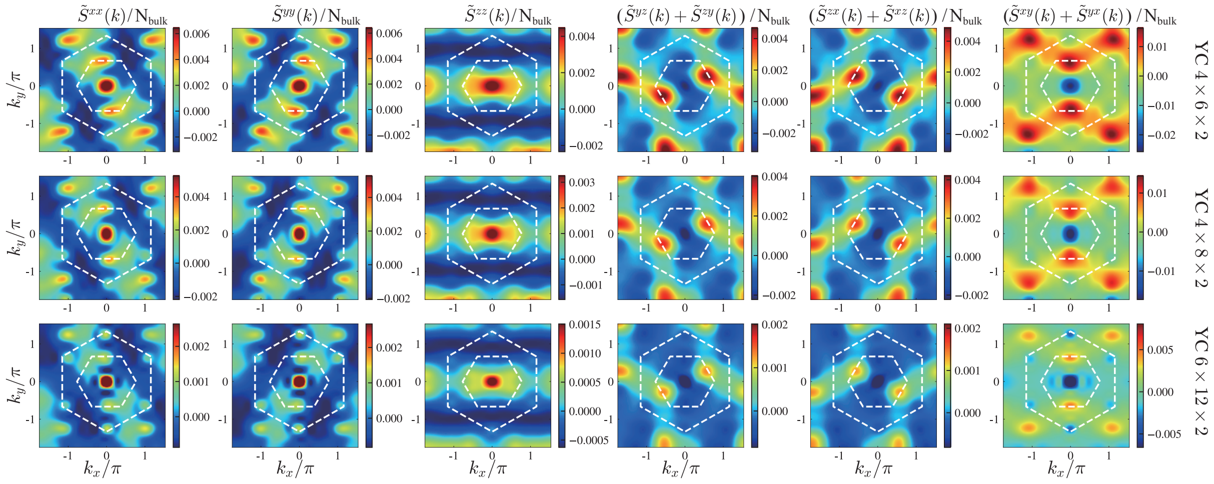

Supplementary Figure 11. **Color maps of the static spin structure factors $\tilde{S}^{\alpha\beta}(\mathbf{k})/N_{\mathrm{bulk}}$.** The results are calculated in the intermediate QSL phase under $\mu_0 H_{[111]} \simeq 78$ T on cylinders of different sizes, obtained by DMRG simulations.

Kitaev interaction (~ -25 meV), which becomes more and more clear as the system cools down near the low-T scale T_1'', around which the brightness at Γ and M points are slightly enhanced due to the strong spin fluctuations. When $T < T_1''$ the Γ peak in the spin structures becomes flattened, yet the stripy background remains distinct, which is in excellent agreement with the DMRG results, supporting strongly the existence of QSL state under high out-of-plane fields.

Ground-state static spin structures under a field of 78 T. In Supplementary Fig. 11 and Supplementary Fig. 12, we show the ground-state static structure factor $\tilde{S}^{\alpha\beta}(\mathbf{k}) = \sum_{j\in \mathrm{bulk},\, j\neq i_0} e^{i\mathbf{k}\cdot(\mathbf{r}_j-\mathbf{r}_{i_0})}(\langle S^\alpha_{i_0} S^\beta_j\rangle - \langle S^\alpha_{i_0}\rangle\langle S^\beta_j\rangle)$ of our α-$\mathrm{RuCl_3}$ model on cylinders of different sizes in the high-field QSL phase (under a magnetic fields of 78 T), obtained by DMRG simulations with bond dimension up to $D = 2048$. Here we fix a central site i_0 and compute the correlations with respect to this reference site, instead of computing the all-to-all correlations, to speed up the computations. From Supplementary Fig. 11, we find strong stripy background in $\tilde{S}^{xx}$, $\tilde{S}^{yy}$, and $\tilde{S}^{zz}$, divided by N_{bulk}, where N_{bulk} is the number of bulk sites (i.e., with the left- and right-most columns of the cylinder skipped), representing intrinsic Kitaev spin liquid characteristics. There also exists a bright Γ point in the BZ, and we depict the profiles with fixed $k_y = 0$ in Supplementary Fig. 12, to check whether the structure factor diverges at any $\mathbf{k}$ point. In Supplementary Fig. 12 we find both the diagonal part $\tilde{S}_{\mathrm{diag}}(\mathbf{k}) = \sum_{\alpha,\beta} \tilde{S}^{\alpha\beta}(\mathbf{k})\delta_{\alpha\beta}$ and the off-diagonal part $\tilde{S}_{\mathrm{off-diag}}(\mathbf{k}) = \sum_{\alpha,\beta} \tilde{S}^{\alpha\beta}(\mathbf{k})(1-\delta_{\alpha\beta})$, when divided by N_{bulk}, are moving towards zero as the system size increases, indicating the absence of spontaneous long-range order in the phase.

Ground-state entanglement scaling. The nature of the intermediate QSL phase can be further characterized by entanglement scaling in the ground state. Here we study the von Neumann entanglement entropy

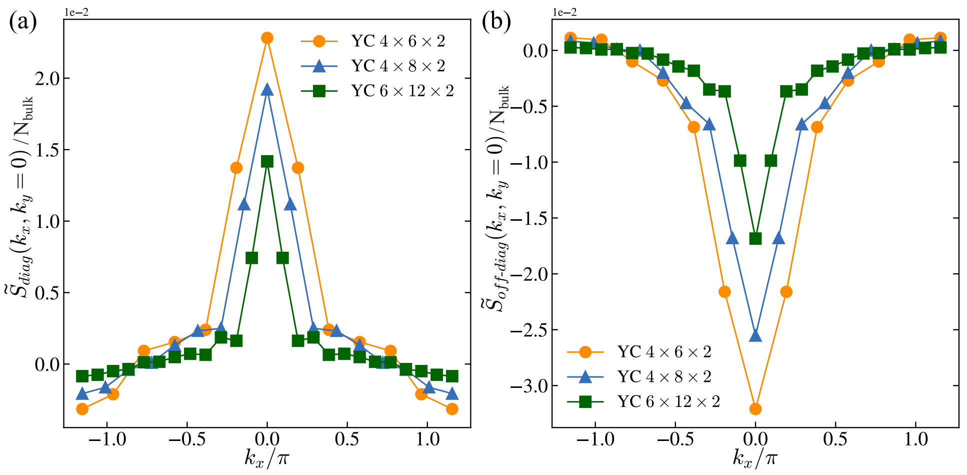

Supplementary Figure 12. **The static spin structure factors cut at $k_y = 0$.** (a) shows $\tilde{S}_{\rm diag}(k_x, k_y = 0)/N_{\rm bulk}$ and (b) $\tilde{S}_{\rm off-diag}(k_x, k_y = 0)/N_{\rm bulk}$ in the intermediate QSL phase under $\mu_0 H_{[111]} \simeq 78$ T on cylinders of different sizes.

S_v. Considering a bipartition of the system into two parts A and B, the reduced density matrix of A can be obtained by tracing out the degrees of freedom of B, i.e., $\rho_{\rm A} = {\rm Tr}_{\rm B}\rho$, where $\rho = |\psi_0\rangle\langle\psi_0|$ is density matrix of the normalized ground state $|\psi_0\rangle$ of the whole system. The von Neumann entropy is defined as

$$S_v = -{\rm Tr}(\rho_{\rm A} {\rm ln} \rho_{\rm A}). \tag{S3}$$

Here, we consider the cut along circumference direction and measure S_v for each cut at l. By virtue of the conformal mapping: $l \to \tilde{l} = (L/\pi)\sin(\pi l/L)$, a central charge c on the open cylindrical geometry can be formally extracted using

$$S_v = \frac{c}{6} \ln \tilde{l} + {\rm const.}, \tag{S4}$$

originally proposed in the (1+1)-D conformal field theory. The prominent “dome”-like S_v curves in Supplementary Fig. 13(a) together with the linear fitting with effective central charge c in Supplementary Fig. 13(b), indicates a gapless intermediate phase. This is consistent with the gapless feature observed in the energy spectra as shown in Fig. 5d and algebraic magnetic specific heat in Fig. 6d of the main text. Furthermore, the effective central charge c increases with the system width W, i.e. the allowed $\hat{y}$ momenta in the Brillouin zone, suggesting the increased gapless modes.

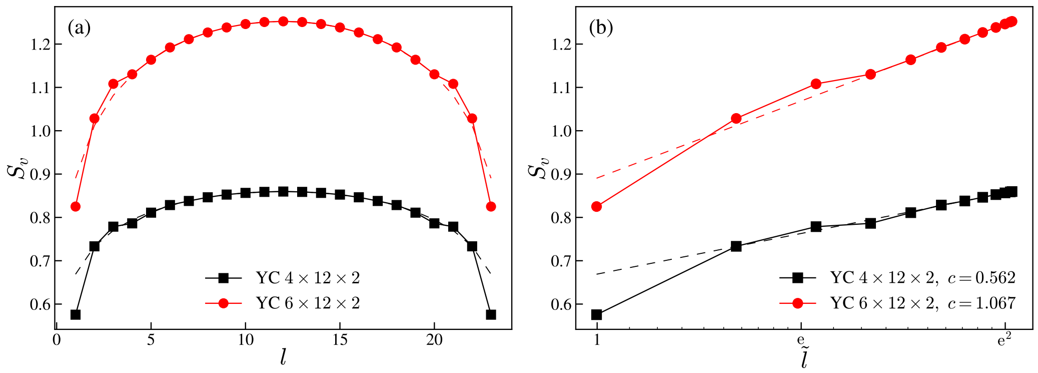

Supplementary Figure 13. **The ground-state entanglement entropy** S_v **in the intermediate phase.** (a) The S_v profile over the system with $\mu_0 H_{[111]} \simeq 78$ T on cylinders of length $L = 12$. (b) S_v versus conformal distance $\tilde{l} \equiv (L/\pi)\sin(\pi l/L)$ in logarithmic scale. The fittings are plotted as dashed lines in both panels, which reveal a central charge c close to 0.5 and 1 for $W = 4$ and 6, respectively.

Supplementary Note 5. Variational Monte Carlo Results

Spin-liquid ansatz based on $D_{3\text{d}} \times Z_2^T$ **symmetry.** We start with the extended Kitaev honeycomb model containing K, J, Γ and Γ' interactions. The most general expression of the mean-field Hamiltonian ansatz [26–28] with nearest-neighbor couplings reads

$$H_{\text{mf}}^{\text{SL}} = \sum_{\langle i,j\rangle \in \alpha\beta(\gamma)} \Big(\text{Tr}\,[U_{ji}^{(0)} \psi_i^\dagger \psi_j] + \text{Tr}\,[U_{ji}^{(1)} \psi_i^\dagger (iR_{\alpha\beta}^\gamma)\psi_j] + \text{Tr}\,[U_{ji}^{(2)} \psi_i^\dagger \sigma^\gamma \psi_j] + \text{Tr}\,[U_{ji}^{(3)} \psi_i^\dagger \sigma^\gamma R_{\alpha\beta}^\gamma \psi_j] + \text{H.c.} \Big) + \sum_i \text{Tr}(\boldsymbol{\lambda}_i \cdot \psi_i \boldsymbol{\tau} \psi_i^\dagger), \tag{S5}$$

where $\psi_i = (C_i\ \bar{C}_i)$, $C_i = (c_{i\downarrow}, c_{i\uparrow})^T$, $\bar{C}_i = (c_{i\downarrow}^\dagger, -c_{i\uparrow}^\dagger)^T$, and $R_{\alpha\beta}^\gamma = -\frac{i}{\sqrt{2}}(\sigma^\alpha + \sigma^\beta)$. Owing to the subtle $SU(2)$ gauge symmetry [29] in the fermionic spinon representation, the particle number constraint $\hat{N}_i = 1$ is extended into the $SU(2)$ gauge invariant three-component form $\text{Tr}(\boldsymbol{\psi}_i \boldsymbol{\tau} \psi_i^\dagger) = 0$ which are ensured by the three Lagrangian multipliers $\lambda^{x,y,z}$, where $\tau^{x,y,z}$ are the generators of the $SU(2)$ gauge group. The matrices $U_{ji}^{(0,1,2,3)}$ can be expanded with the identity matrix and $\tau^{x,y,z}$, where the expanding coefficients form a subset of $\boldsymbol{R}$. For the present model, the $SU(2)$ gauge symmetry breaks down to its subgroup Z_2 (which is called the invariant gauge group, IGG) and the resultant quantum spin liquids are Z_2 QSLs.

A QSL ground state preserves the whole space group symmetry whose point group is $D_{3\text{d}} \times Z_2^T$. However, the symmetry group of a spin liquid mean-field Hamiltonian is the projective symmetry group (PSG) [30, 31] whose group elements are space group operations followed by $SU(2)$ gauge transformations. Although there are more than 100 classes of PSGs for Z$_2$ QSLs, here we adopt the PSG that describes the symmetry of the mean-field Hamiltonian of the pure Kitaev model (called Kitaev PSG). Under this

constraint, the allowed mean-field Hamiltonian contains the following parameters.

Firstly, recalling that in KSL the Kitaev interactions are decoupled in a way that the c Majorana fermions are not mixed with the b^γ Majorana fermions, we have

$$\begin{aligned} H_{\text{mf}}^{K} &= \sum_{\langle i,j\rangle\in\alpha\beta(\gamma)} \rho_a(ic_ic_j) + \rho_c(ib_i^\gamma b_j^\gamma) \\ &= \sum_{\langle i,j\rangle\in\alpha\beta(\gamma)} \Big(i\rho_a \text{Tr}\big(\psi_i^\dagger\psi_j + \tau^x\psi_i^\dagger\sigma^x\psi_j + \tau^y\psi_i^\dagger\sigma^y\psi_j + \tau^z\psi_i^\dagger\sigma^z\psi_j\big) \\ &\quad + i\rho_c \text{Tr}\big(\psi_i^\dagger\psi_j + \tau^\gamma\psi_i^\dagger\sigma^\gamma\psi_j - \tau^\alpha\psi_i^\dagger\sigma^\alpha\psi_j - \tau^\beta\psi_i^\dagger\sigma^\beta\psi_j\big) + \text{H.c.}\Big) \end{aligned} \tag{S6}$$

Similarly, the Γ interactions are decoupled as

$$\begin{aligned} H_{\text{mf}}^{\Gamma} &= \sum_{\langle i,j\rangle\in\alpha\beta(\gamma)} i\rho_d(b_i^\alpha b_j^\beta + b_i^\beta b_j^\alpha) \\ &= \sum_{\langle i,j\rangle\in\alpha\beta(\gamma)} \Big(i\rho_d \text{Tr}\big(\tau^\alpha\psi_i^\dagger\sigma^\beta\psi_j + \tau^\beta\psi_i^\dagger\sigma^\alpha\psi_j\big) + \text{H.c.}\Big), \end{aligned} \tag{S7}$$

and the Γ' interactions are decoupled as

$$\begin{aligned} H_{\text{mf}}^{\Gamma'} &= \sum_{\langle i,j\rangle\in\alpha\beta(\gamma)} i\rho_f(b_i^\alpha b_j^\gamma + b_i^\gamma b_j^\alpha + b_i^\beta b_j^\gamma + b_i^\gamma b_j^\beta) \\ &= \sum_{\langle i,j\rangle\in\alpha\beta(\gamma)} \Big(i\rho_f \text{Tr}\big(\tau^\alpha\psi_i^\dagger\sigma^\gamma\psi_j + \tau^\gamma\psi_i^\dagger\sigma^\alpha\psi_j + \tau^\beta\psi_i^\dagger\sigma^\gamma\psi_j + \tau^\gamma\psi_i^\dagger\sigma^\beta\psi_j\big) + \text{H.c.}\Big). \end{aligned} \tag{S8}$$

Comparing with the general form Supplementary Eq. (S5), the decouplings expressed in Supplementary Eqs. (S6), (S7) and (S8) contribute the terms $\tilde{U}_{ji}^{(0)} = i(\rho_a + \rho_c)$, $\tilde{U}_{ji}^{(1)} = i(\rho_a - \rho_c + \rho_d + 2\rho_f)(\tau^\alpha + \tau^\beta)$, $\tilde{U}_{ji}^{(2)} = i(\rho_a + \rho_c)\tau^\gamma + i\rho_f(\tau^\alpha + \tau^\beta)$, $\tilde{U}_{ji}^{(3)} = i(\rho_c - \rho_a - \rho_d)(\tau^\alpha - \tau^\beta)$, to the coefficients $U_{ji}^{(m)}$, in which j and i specify γ.

Secondly, we consider a more general mean-field decoupling which means c Majorana fermions can mix with b^γ Majorana fermions. In addition, the most general coefficients preserving the C_3 rotation symmetry (in the PSG sense) also contain multiples of the uniform (I) and $\tau^x + \tau^y + \tau^z$ gauge components, $\tilde{\tilde{U}}_{ji}^{(0)} = i\eta_0 + \eta_1(\tau^x + \tau^y + \tau^z)$, $\tilde{\tilde{U}}_{ji}^{(1)} = \eta_2 + i\eta_3(\tau^x + \tau^y + \tau^z)$, $\tilde{\tilde{U}}_{ji}^{(2)} = \eta_4 + i\eta_5(\tau^x + \tau^y + \tau^z)$, $\tilde{\tilde{U}}_{ji}^{(3)} = \eta_6 + i\eta_7(\tau^x + \tau^y + \tau^z)$. If the full symmetry group, $G = D_{3\text{d}} \times Z_2^T$, is preserved, then only three parameters η_0, η_3, and η_5 are allowed. Thus a spin-liquid ansatz that preserves the full Kitaev PSG contains the variables

$U_{ji}^{(m)} = \tilde{U}_{ji}^{(m)} + \tilde{\tilde{U}}_{ji}^{(m)}$, namely

$$\begin{aligned} U_{ji}^{(0)} &= i(\rho_a + \rho_c) + i\eta_0, \\ U_{ji}^{(1)} &= i(\rho_a - \rho_c + \rho_d + 2\rho_f)(\tau^\alpha + \tau^\beta) + i\eta_3(\tau^x + \tau^y + \tau^z), \\ U_{ji}^{(2)} &= i(\rho_a + \rho_c)\tau^\gamma + i\rho_f(\tau^\alpha + \tau^\beta) + i\eta_5(\tau^x + \tau^y + \tau^z), \\ U_{ji}^{(3)} &= i(\rho_c - \rho_a - \rho_d)(\tau^\alpha - \tau^\beta), \end{aligned} \tag{S9}$$

with seven real parameters, ρ_a, ρ_c, ρ_d, ρ_f, η_0, η_3 and η_5.

Magnetically ordered states. Because fermions do not condense alone to form a magnetic order, we introduce the classical order under single-$\boldsymbol{Q}$ approximation [32] to describe the magnetic order of the spin-symmetry-breaking phases of the K-J-Γ-Γ' model.

$$\boldsymbol{M}_i = M\{\sin\phi[\hat{\boldsymbol{e}}_x \cos(\boldsymbol{Q}\cdot\boldsymbol{r}_i) + \hat{\boldsymbol{e}}_y \sin(\boldsymbol{Q}\cdot\boldsymbol{r}_i)] + \cos\phi\,\hat{\boldsymbol{e}}_z\},$$

where $\boldsymbol{Q}$ is the ordering momentum, $\hat{\boldsymbol{e}}_{x,y,z}$ are the local spin axes (not to be confused with the global spin axes), and ϕ is the canting angle. $\pi/2 - \phi$ describes the angle by which the spins deviate from the plane spanned by $\hat{\boldsymbol{e}}_x$ and $\hat{\boldsymbol{e}}_y$. The classical ground state is obtained by minimizing the energy of the trial states.

In our VMC calculations, the static order is treated as a background field coupling to the spins as site-dependent Zeeman field, hence the complete mean-field Hamiltonian for the K-J-Γ-Γ' model reads

$$H_{\text{mf}}^{\text{total}} = H_{\text{mf}}^{\text{SL}} - \tfrac{1}{2}\sum_i (\boldsymbol{M}_i \cdot C_i^\dagger \boldsymbol{\sigma} C_i + \text{H.c.}) \tag{S10}$$

The ordering momentum $\boldsymbol{Q}$ of $\boldsymbol{M}_i$ in VMC is adopted from the classical ground state or the classical metastable states (depending on the energy of the projected state). For a given $\boldsymbol{Q}$, the local axes $\hat{\boldsymbol{e}}_{x,y,z}$ are fixed as they are in the classical state, M and ϕ are treated as variational parameters. The amplitude of the static magnetic order M we compute includes quantum corrections.

Finally, since the magnetic order essentially breaks the C_3 symmetry, the parameter η_7 and an additional parameter θ describing the ratio of certain parameters in the z-bond and x-(y-) bonds are allowed (similar to the model discussed in Ref. [33]).

Field-induced chiral spin liquid. By comparing various ansatzes, including the spin-liquid and magnetically ordered states, our VMC calculations reveals an exotic phase under the out-of-plane magnetic field $\boldsymbol{B} \equiv \mu_0 H_{[111]}$. As the zigzag order being suppressed by a large field, an intermediate chiral spin liquid (CSL) phase with Chern number $\nu = 2$ emerges before the system enters the polarized phase. The phase

transition between the zigzag ordered state and the the CSL phase is of first order. According to Kitaev's seminal work [34], the CSL is an Abelian phase whose quasiparticle excitations include $a, \bar{a}, \varepsilon, 1$, where 1 denotes the vacuum, ε is the fermion, a and $\bar{a}$ are two types of vortices with topological spin $e^{i\pi/4}$. The fusion rules are $\varepsilon \times \varepsilon = 1, a \times \bar{a} = 1, a \times \varepsilon = \bar{a}, \bar{a} \times \varepsilon = a, \bar{a} \times \bar{a} = \varepsilon$. The edge of the CSL state is gapless and contains 2 branches of chiral Majorana excitations, each branch carries a chiral central charge of $1/2$. Therefore, the thermal Hall conductance is integer quantized, with $\kappa_{xy}/T = \pi k_B^2/6h$ at low temperature.

In the following, we discuss whether the field-induced Z_2 CSL is topologically nontrivial or not. The confinement or deconfinement of the Z_2 gauge field is reflected in the ground state degeneracy (GSD) of the Gutzwiller projected state when placed on a torus. Therefore, we calculate the density matrix of the projected states from the wave-function overlap $\rho_{\alpha\beta} = \langle P_G \psi_\alpha | P_G \psi_\beta \rangle = \rho^*_{\beta\alpha}$, where $\alpha, \beta \in \{++, +-, -+, --\}$ are the boundary conditions ($+$ stands for the periodic boundary condition and $-$ for antiperiodic boundary condition) along $\boldsymbol{a}_1$ and $\boldsymbol{a}_2$ direction, respectively. If ρ has only one significant eigenvalue, with the others vanishingly small, then the GSD is 1, indicating that the Z_2 gauge field is confined. Otherwise, for ρ with more than one (near-degenerate) nonzero eigenvalues, then the GSD is nontrivial and hence the Z_2 gauge fluctuations are deconfined. In the VMC calculations, we find the field-induced CSL (with Chern number $\nu = 2$) is deconfined, with GSD equals 4 (the eigenvalues of the overlap matrix ρ are given by $0.5915, 0.8066, 1.0419, 1.5600$ under $g_{c^*}\mu_B B/|K| = 0.52$ for a system on an $8\times8\times2$ torus). Actually, we have performed a finite-size scaling calculation (not shown), which indeed indicates that the GSD of this state is 4 in the large-size limit. Indeed, such a GSD matches the number of topologically distinct quasiparticle types.

---

[1] Y. Kubota, H. Tanaka, T. Ono, Y. Narumi, and K. Kindo, "Successive magnetic phase transitions in α-RuCl$_3$: XY-like frustrated magnet on the honeycomb lattice," Phys. Rev. B **91**, 094422 (2015).

[2] S.-H. Do, S.-Y. Park, J. Yoshitake, J. Nasu, Y. Motome, Y. S. Kwon, D. T. Adroja, D. J. Voneshen, K. Kim, T.-H. Jang, J.-H. Park, K.-Y. Choi, and S. Ji, "Majorana fermions in the Kitaev quantum spin system α-RuCl$_3$," Nat. Phys. **13**, 1079 (2017).

[3] S. Widmann, V. Tsurkan, D. A. Prishchenko, V. G. Mazurenko, A. A. Tsirlin, and A. Loidl, "Thermodynamic evidence of fractionalized excitations in α-RuCl$_3$," Phys. Rev. B **99**, 094415 (2019).

[4] S. M Winter, A. A Tsirlin, M. Daghofer, J. van den Brink, Y. Singh, P. Gegenwart, and R. Valentí, "Models and materials for generalized Kitaev magnetism," J. Phys.: Condens. Matter **29**, 493002 (2017).

[5] S. M. Winter, K. Riedl, P. A. Maksimov, A. L. Chernyshev, A. Honecker, and R. Valentí, "Breakdown of magnons in a strongly spin-orbital coupled magnet," Nat. Commun. **8**, 1152 (2017).

[6] S. M. Winter, K. Riedl, D. Kaib, R. Coldea, and R. Valentí, "Probing α-RuCl$_3$ beyond magnetic order: Effects

of temperature and magnetic field," Phys. Rev. Lett. **120**, 077203 (2018).

[7] J. S. Gordon, A. Catuneanu, E. S. Sørensen, and H.-Y. Kee, "Theory of the field-revealed Kitaev spin liquid," Nat. Commun. **10**, 2470 (2019).

[8] C. Hickey and S. Trebst, "Emergence of a field-driven U(1) spin liquid in the Kitaev honeycomb model," Nat. Commun. **10**, 530 (2019).

[9] P. Laurell and S. Okamoto, "Dynamical and thermal magnetic properties of the Kitaev spin liquid candidate α-$RuCl_3$," npj Quantum Mater. **5**, 2 (2020).

[10] P. Lampen-Kelley, S. Rachel, J. Reuther, J.-Q. Yan, A. Banerjee, C. A. Bridges, H. B. Cao, S. E. Nagler, and D. Mandrus, "Anisotropic susceptibilities in the honeycomb Kitaev system α-$RuCl_3$," Phys. Rev. B **98**, 100403 (2018).

[11] J. A. Sears, L. E. Chern, S. Kim, P. J. Bereciartua, S. Francoual, Y. B. Kim, and Y.-J. Kim, "Ferromagnetic Kitaev interaction and the origin of large magnetic anisotropy in α-$RuCl_3$," Nat. Phys. **16**, 837–840 (2020).

[12] H. Li, D.-W. Qu, H.-K. Zhang, Y.-Z. Jia, S.-S. Gong, Y. Qi, and W. Li, "Universal thermodynamics in the Kitaev fractional liquid," Phys. Rev. Research **2**, 043015 (2020).

[13] S. Yu, Y. Gao, B.-B. Chen, and W. Li, "Learning the effective spin Hamiltonian of a quantum magnet," arXiv:2011.12282 (2020).

[14] S. M. Winter, Y. Li, H. O. Jeschke, and R. Valentí, "Challenges in design of Kitaev materials: Magnetic interactions from competing energy scales," Phys. Rev. B **93**, 214431 (2016).

[15] L. Wu, A. Little, E. E. Aldape, D. Rees, E. Thewalt, P. Lampen-Kelley, A. Banerjee, C. A. Bridges, J.-Q. Yan, D. Boone, S. Patankar, D. Goldhaber-Gordon, D. Mandrus, S. E. Nagler, E. Altman, and J. Orenstein, "Field evolution of magnons in α-$RuCl_3$ by high-resolution polarized terahertz spectroscopy," Phys. Rev. B **98**, 094425 (2018).

[16] J. Cookmeyer and J. E. Moore, "Spin-wave analysis of the low-temperature thermal Hall effect in the candidate Kitaev spin liquid α-$RuCl_3$," Phys. Rev. B **98**, 060412 (2018).

[17] H.-S. Kim and H.-Y. Kee, "Crystal structure and magnetism in α-$RuCl_3$: An ab initio study," Phys. Rev. B **93**, 155143 (2016).

[18] T. Suzuki and S.-i. Suga, "Erratum: Effective model with strong Kitaev interactions for α-$RuCl_3$ [Phys. Rev. B 97, 134424 (2018)]," Phys. Rev. B **99**, 249902 (2019).

[19] K. Ran, J. Wang, W. Wang, Z.-Y. Dong, X. Ren, S. Bao, S. Li, Z. Ma, Y. Gan, Y. Zhang, J. T. Park, G. Deng, S. Danilkin, S.-L. Yu, J.-X. Li, and J. Wen, "Spin-wave excitations evidencing the Kitaev interaction in single crystalline α-$RuCl_3$," Phys. Rev. Lett. **118**, 107203 (2017).

[20] W. Wang, Z.-Y. Dong, S.-L. Yu, and J.-X. Li, "Theoretical investigation of magnetic dynamics in α-$RuCl_3$," Phys. Rev. B **96**, 115103 (2017).

[21] I. O. Ozel, C. A. Belvin, E. Baldini, I. Kimchi, S.-H. Do, K.-Y. Choi, and N. Gedik, "Magnetic field-dependent low-energy magnon dynamics in α-$RuCl_3$," Phys. Rev. B **100**, 085108 (2019).

[22] J. A. Sears, M. Songvilay, K. W. Plumb, J. P. Clancy, Y. Qiu, Y. Zhao, D. Parshall, and Y.-J. Kim, "Magnetic order in α-$RuCl_3$: A honeycomb-lattice quantum magnet with strong spin-orbit coupling," Phys. Rev. B **91**,

144420 (2015).

[23] A. Banerjee, J. Yan, J. Knolle, C. A. Bridges, M. B. Stone, M. D. Lumsden, D. G. Mandrus, D. A. Tennant, R. Moessner, and S. E. Nagler, "Neutron scattering in the proximate quantum spin liquid α-RuCl$_3$," Science **356**, 1055–1059 (2017).

[24] A. Banerjee, P. Lampen-Kelley, J. Knolle, C. Balz, A. Aczel, B. Winn, Y. Liu, D. Pajerowski, J. Yan, C. A. Bridges, A. T. Savici, B. C. Chakoumakos, M. D. Lumsden, D. A. Tennant, R. Moessner, D. G. Mandrus, and S. E. Nagler, "Excitations in the field-induced quantum spin liquid state of α-RuCl$_3$," npj Quantum Mater. **3**, 8 (2018).

[25] T. Yokoi, S. Ma, Y. Kasahara, S. Kasahara, T. Shibauchi, N. Kurita, H. Tanaka, J. Nasu, Y. Motome, C. Hickey, S. Trebst, and Y. Matsuda, "Half-integer quantized anomalous thermal Hall effect in the Kitaev material α-RuCl$_3$," arXiv:2001.01899 (2020).

[26] J. Wang, B. Normand, and Z.-X. Liu, "One proximate Kitaev spin liquid in the $K-J-\Gamma$ model on the honeycomb lattice," Phys. Rev. Lett. **123**, 197201 (2019).

[27] J. Wang, Q. Zhao, X. Wang, and Z.-X. Liu, "Multinode quantum spin liquids on the honeycomb lattice," Phys. Rev. B **102**, 144427 (2020).

[28] Z.-X. Liu and B. Normand, "Dirac and Chiral Quantum Spin Liquids on the Honeycomb Lattice in a Magnetic Field," Phys. Rev. Lett. **120**, 187201 (2018).

[29] I. Affleck, Z. Zou, T. Hsu, and P. W. Anderson, "SU(2) gauge symmetry of the large-U limit of the Hubbard model," Phys. Rev. B **38**, 745 (1988).

[30] X.-G Wen, "Quantum Orders and Symmetric Spin Liquids," Phys. Rev. B **65**, 165113 (2002).

[31] Y.-Z. You, I. Kimchi, and A. Vishwanath, "Doping a spin-orbit Mott Insulator: Topological Superconductivity from the Kitaev-Heisenberg Model and possible application to (Na$_2$/Li$_2$)IrO$_3$," Phys. Rev. B **86**, 085145 (2012).

[32] J. G. Rau, E. K.-H. Lee, and H.-Y. Kee, "Generic Spin Model for the Honeycomb Iridates beyond the Kitaev Limit," Phys. Rev. Lett. **112**, 077204 (2014).

[33] J. Wang and Z.-X. Liu, "Symmetry-protected gapless spin liquids on the strained honeycomb lattice," Phys. Rev. B **102**, 094416 (2020).

[34] A. Kitaev, "Anyons in an exactly solved model and beyond," Ann. Phys. **321**, 2–111 (2006).
